# Synthesis of pyrrolo[3,2-*d*]pyrimidine-2,4(3*H*)-diones by domino C–N coupling/hydroamination reactions

**DOI:** 10.3762/bjoc.21.82

**Published:** 2025-05-22

**Authors:** Ruben Manuel Figueira de Abreu, Robin Tiedemann, Peter Ehlers, Peter Langer

**Affiliations:** 1 Universität Rostock, Institut für Chemie, Albert-Einstein-Str. 3a, 18059 Rostock, Germany; 2 Leibniz Institut für Katalyse an der Universität Rostock e. V., Albert-Einstein-Str. 29a, 18059 Rostock, Germany

**Keywords:** alkynes, catalysis, cyclizations, domino reactions, heterocycles

## Abstract

A variety of pyrrolo[3,2-*d*]pyrimidine-2,4(3*H*)-diones were prepared by a combination of Sonogashira reaction and subsequent cyclization by domino C–N coupling/hydroamination reaction. The optical properties (UV–vis absorption and fluorescence) depend on the substitution pattern of the compounds.

## Introduction

**Figure 1:**
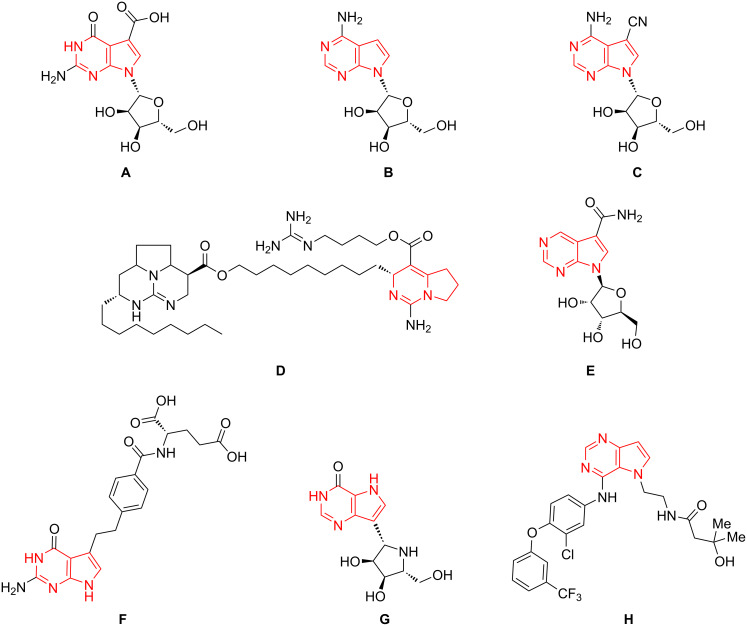
Development of drugs based on pyrrolopyrimidines: **A**: Cadeguomycin. **B**: Tubercidin. **C**: Toyocamycin. **D**: Batzelladine A. **E**: Sangivamycin. **F**: Pemetrexed. **G**: Immucillin H. **H**: TAK-285 (tyrosine kinase inhibitor).

Pyrimidines and purines are one of the most important heterocyclic compounds with prevalent biological functions. Both are found in nucleosides and their corresponding polymeric DNA and RNA, and hence are vital for life on Earth. The importance of these heteroaromatic derivatives has stimulated tremendous investigation towards the understanding of genetic information transmission as well as the synthesis of novel derivatives for medicinal applications [1–15]. For instance, deazapurines represent heterocyclic fused pyrimidine bases which have found special attention, due to their widespread occurrence in natural alkaloids exhibiting various biological properties. For example, cadeguomycin (**A**), tubercidin (**B**), and toyocamycin (**C**) show antibiotic properties, while batzelladine A (**D**), isolated from a Bahamian sponge, possesses anti-HIV and cancerostatic activities. Antiviral properties have been identified for sangivamycin (**E**), which was isolated from *Streptomyces rimosus* (Figure 1) [16–21].

Consequently, several pyrrolopyrimidines have been synthesized to develop novel pharmaceuticals with improved biological properties. For instance, pemetrexed (**F**) is administered during palliative chemotherapy for advanced lung cancers [12]. Immucillin H (**G**) is currently in late clinical phases for the treatment of haematologic diseases, such as acute T-cell leukaemia, and TAK-285 (**H**) is a promising HER2/EGFR inhibitor which has been tested in a phase 1 trial on humans as an anticancer agent (Figure 1) [16]. Given the significance of deazapurines as biologically active lead compounds [22], we developed a new methodology for the synthesis of uracil-based pyrrolopyrimidine derivatives. The envisioned methodology combines a Sonogashira–Hagihara reaction and a one-pot process comprising a Buchwald–Hartwig coupling reaction followed by a hydroamination [23–24].

## Results and Discussion

**Scheme 1:**
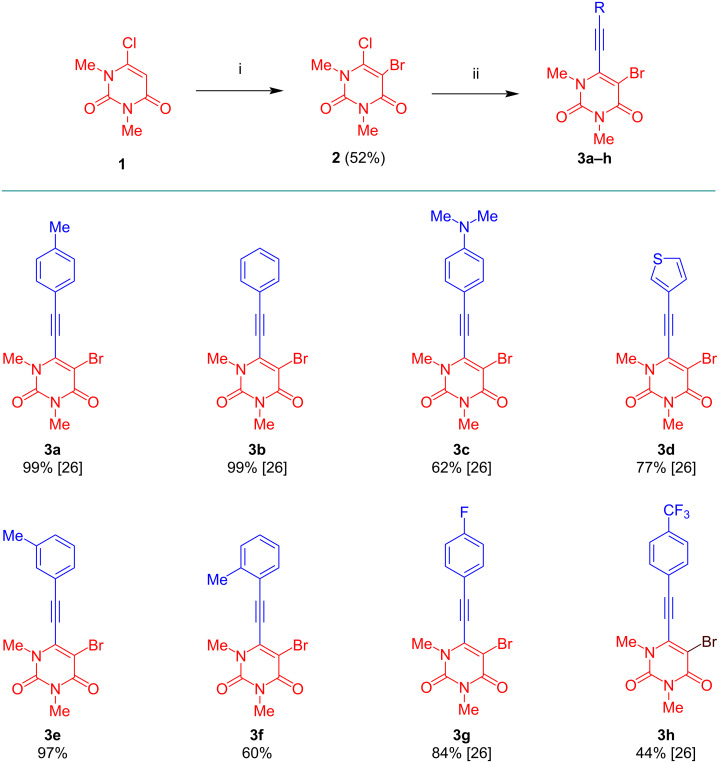
Synthesis of **3a**–**h**. Conditions: i) Br_2_ (1.0 equiv), Ac_2_O (1.5 equiv), AcOH, 25 °C, 1 h [25]; ii) aryl acetylene (1.2 equiv), Pd(PPh_3_)Cl_2_ (5 mol %), CuI (5 mol %), NEt_3_ (10 equiv), DMSO, 25 °C, 6 h [26]. Yields of isolated products.

The bromination of 6-chloro-1,3-dimethyluracil (**1**) afforded, following a known procedure [25], 5-bromo-6-chloro-1,3-dimethyluracil (**2**) in 52% yield (Scheme 1). We previously reported Sonogashira reactions of the latter with various alkynes to give products **3a**–**d**,**g**,**h** [26–27]. In this work, we extended the scope and prepared novel derivatives **3e** and **3f**. A nearly quantitative yield was obtained for product **3e** derived from 3-tolylacetylene. However, the yield for compound **3f** dropped to 60% because of the more sterically hindered 2-tolylacetylene.

**Scheme 2:**
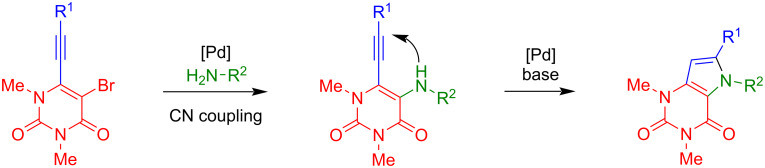
C–N cross-coupling/hydroamination reaction.

The domino C–N cross-coupling/hydroamination reaction of **3a–h** with various anilines was studied next (Scheme 2) [28–29].

The conditions were optimized for the reaction of **3a** with *p*-toluidine to give pyrrolo[3,2-*d*]pyrimidine-2,4(3*H*)-dione **4a** (Table 1). For the first experiment, we chose Pd(OAc)_2_ (5 mol %) as the catalyst, XPhos (5 mol %) as the ligand and K_3_PO_4_ (3 equiv) as the base in DMA (100 °C, 15 hours), which previously proved to be efficient for related transformations [28–29]. However, only a yield of 15% of the desired product **4a** was obtained after stirring for 15 hours, due to low conversion of the starting material. Subsequently, different mono- and bidentate ligands were tested. DPEphos was found to be the most potent ligand, leading to 43% isolated yield with full conversion of starting material and different byproducts derived from decomposition. Interestingly, no conversion of the starting material could be observed with the other ligands. In the following, different solvents, temperatures and bases were tested, but did not result in a further improvement of the yield.

**Table 1:** Optimization of the synthesis of **4a**.

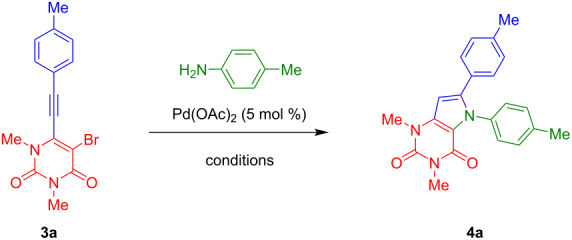					

Entry	Ligand (5 mol %)	Base (3 equiv)	Solvent	Temp (°C)	Yield (%)

1	XPhos	K_3_PO_4_	DMA	100	15
2	Xantphos	K_3_PO_4_	DMA	100	–
3	DPEphos	K_3_PO_4_	DMA	100	43
4	Dppf	K_3_PO_4_	DMA	100	–
5	CataCXium A	K_3_PO_4_	DMA	100	–
6	Ruphos	K_3_PO_4_	DMA	100	–
7	P(*t*-Bu)_3_·HBF_4_	K_3_PO_4_	DMA	100	–
8	DPEphos	K_3_PO_4_	toluene	100	25
9	DPEphos	K_3_PO_4_	1,4-dioxane	100	34
10	DPEphos	NaO*t*-Bu	DMA	100	14
11	DPEphos	KO*t*-Bu	DMA	100	15
12	DPEphos	Cs_2_CO_3_	DMA	100	9
13	DPEphos	KN(SiMe_3_)_2_	DMA	100	–
14	DPEphos	K_3_PO_4_	DMA	120	15

**Scheme 3:**
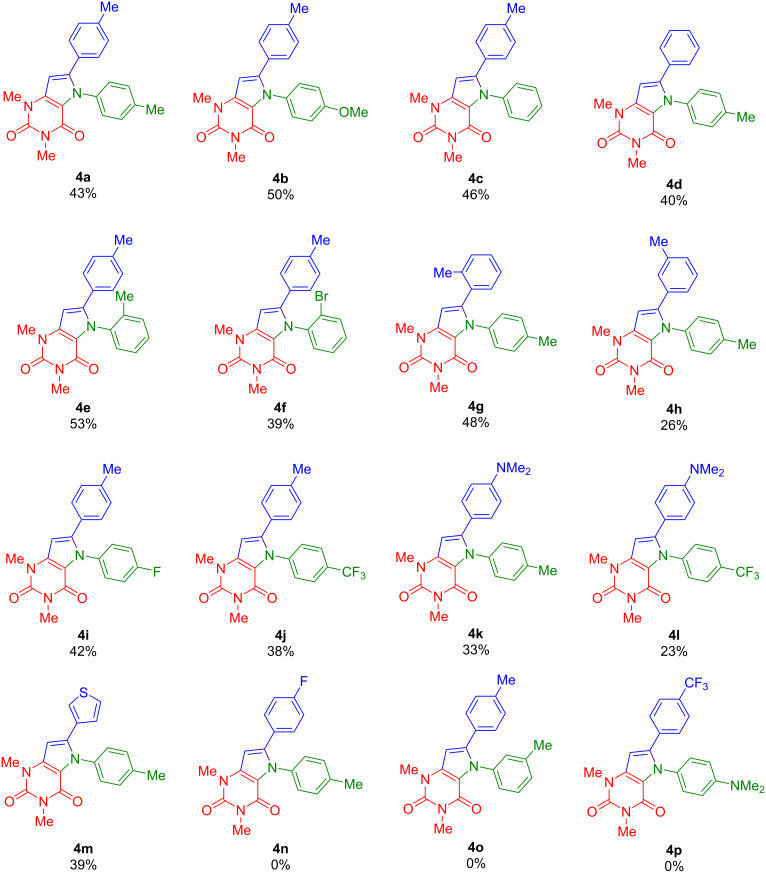
Synthesis of **4a**–**m**. Conditions: Pd(OAc)_2_ (5 mol %), DPEphos (5 mol %), K_3_PO_4_ (3 equiv), DMA, 100 °C, 15 h. Yields of isolated products.

With the optimized conditions in hand, the scope of the reaction was studied. The cyclization of alkynylated uracils **3a**–**h** with various anilines afforded pyrrolo[3,2-*d*]pyrimidine-2,4(3*H*)-diones **4a**–**m** in moderate to good yields (Scheme 3). Various functional groups attached to the aniline, such as OMe, F, CF_3_, Me, and Br, were tolerated and did not greatly differ in terms of yield. With respect to substituents located at the alkynyl moiety, diminished yields were obtained for *N,N*-dimethylaminophenyl-substituted derivatives **4k** and **4l** and for *m*-tolyl-substituted compound **4h**. No conversion was observed for starting materials **3g**,**h** containing electron-withdrawing substituents located at the phenylacetylene moiety (products **4n,p**). In addition, no conversion was observed for 3-methylaniline (product **4o**).

**Figure 2:**
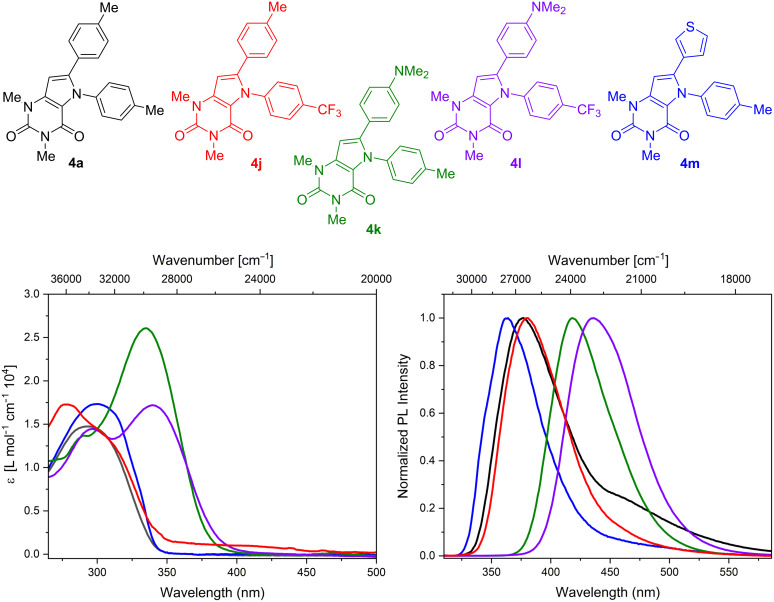
UV–vis absorption (left) and emission (right, λ_ex_ = 300 nm) spectra of compounds **4a**, **4j**, **4k**, **4l**, and **4m** in dichloromethane (*c* = 1·10^−5^ M).

The photophysical properties of selected pyrrolo[3,2-*d*]pyrimidine-2,4(3*H*)-diones **4** were investigated by steady-state absorption and photoluminescence spectroscopy (Figure 2, Table 2). The wavelength and intensity of absorption and emission depended on the substitution pattern. The methyl-substituted compound **4a** showed one broad absorption band at ≈290 nm. A similar absorption feature, but slightly bathochromically shifted, was observed for the thienyl-substituted derivative **4m**. The strongly electron-donating *N,N*-dimethylaminophenyl group (products **4k**,**l**) resulted in segmentation into two absorption bands accompanied by a strongly red-shifted lower energy absorption band. With respect to the phenyl group attached to the pyrrole nitrogen, the presence of the electron-withdrawing CF_3_ group led to a slight hypsochromic shift (**4j**). In the case of compound **4l**, containing a 4-trifluoromethylphenyl group attached to the nitrogen, no significant shift of the absorption was observed as compared to compound **4k** containing a 4-tolyl group. However, the presence of a CF_3_ group resulted in a reduction of the extinction coefficient of the absorption band.

**Table 2:** Photophysical data of **4a**, **4j**, **4k**, **4l**, and **4m** in dichloromethane (*c* = 1·10^−5^ M) at 20 °C.

	**4a**	**4j**	**4k**	**4l**	**4m**

λ_1,abs_ (nm) ε_λ1_·10^4^ (M^−1^ cm^−1^)	293 1.5	278 1.7	289 1.4	297 1.4	299 1.7
λ_2,abs_ (nm) ε_λ2_·10^4^ (M^−1^ cm^−1^)			335 2.6	339 1.7	
λ_1,em_^400^ (nm) λ_2,em_^400^ (nm)	377 462^a^	380	418	436	364
Φ^b^	9%	0.1%	83%	71%	4%

^a^Shoulder in the spectrum. ^b^For the excitation wavelength λ_ex_ = 300 nm; fluorescence standard: quinine sulfate in H_2_SO_4_ (0.05 M) (Φ = 0.52) [30].

The emission spectrum of **4a** consisted of a strong emission band at 377 nm with a weak shoulder expiring to higher wavelengths. However, this shoulder was less distinct for compounds **4m** and **4j** and not detectable for **4k** and **4l**, containing the *N,N*-dimethylaminophenyl group. In contrast to the absorption spectra, in the case of the emission spectra, the presence of a thienyl substituent (compound **4m**) led to a hypsochromic shift, while the emission band of **4j** was slightly red-shifted. The *N,N*-dimethylaminophenyl-substituted compounds **4k** and **4l** showed the strongest bathochromic shifts of the emission spectra, which might be due to the occurrence of donor–acceptor interactions between the electron-deficient uracil and the amino group. The corresponding fluorescence quantum yields were also strongly affected by the substitution pattern of the pyrrolouracils. Compounds **4k** and **4l** show very high fluorescence quantum yields of 83% and 71%, respectively, what might be reasoned by the strong donor ability of the NMe_2_-functional groups of those compounds. In contrast, compounds **4a** and **4m** showed only weak fluorescence with quantum yields of 9 and 4%, respectively. Compound **4j** exhibited almost no emission (Φ = 0.1%). [30].

## Conclusion

In summary, we developed a new methodology for the synthesis of pyrrolo[3,2-*d*]pyrimidine-2,4(3*H*)-diones based on a domino C–N coupling/hydroamination reaction of readily available alkynylated uracils with anilines. The optimized reaction conditions allowed for the employment of various functional groups. The products revealed fluorescence properties which were influenced by the substitution pattern. Electron-donating *N,N*-dimethylaminophenyl substituents led to bathochromically shifted absorption and emission spectra accompanied by strongly elevated fluorescence quantum yields (up to 83%). Further studies will be devoted to the synthesis of novel polycyclic uracil derivatives with potential biological activities.

## Supporting Information

File 1 Experimental section.

## Data Availability

All data that supports the findings of this study is available in the published article and/or the supporting information of this article.
