# Supplementary material for: Synthesis of pyrrolo[3,2-d]pyrimidine-2,4(3H)-diones by domino C–N coupling/hydroamination reactions
Source: Beilstein J Org Chem. 2025 May 22;21:1010–7. doi: 10.3762/bjoc.21.82 (PMC12117214; doi:10.3762/bjoc.21.82)
Supplement: File 1 — Experimental section. [file Beilstein_J_Org_Chem-21-1010-s001.pdf]

BEILSTEIN JOURNAL OF ORGANIC CHEMISTRY

Supporting Information

for

Synthesis of pyrrolo[3,2-*d*]pyrimidine-2,4(3*H*)-diones by domino C–N coupling/hydroamination reactions

Ruben Manuel Figueira de Abreu, Robin Tiedemann, Peter Ehlers and Peter Langer

Beilstein J. Org. Chem. **2025,** *21,* 1010–1017. doi:10.3762/bjoc.21.82

Experimental section

License and Terms: This is a supporting information file under the terms of the Creative Commons Attribution License (https://creativecommons.org/licenses/by/4.0). Please note that the reuse, redistribution and reproduction in particular requires that the author(s) and source are credited and that individual graphics may be subject to special legal provisions.
The license is subject to the *Beilstein Journal of Organic Chemistry* terms and conditions: (https://www.beilstein-journals.org/bjoc/terms)

Table of contents

Experimental section S2

^{1}H, ^{13}C, ^{19}F NMR spectra S8

References S26

Experimental section

General information

Nuclear magnetic resonance spectra ($^{1}H/^{13}C/^{19}F$ NMR) were recorded on a Bruker AVANCE 300 III, 250 II, or 500. The analysed chemical shifts δ are referenced to the residual solvent signals of the deuterated solvents $CDCl_3$ (δ = 7.26 ppm/77.16 ppm). Multiplicities due to spin–spin correlation are reported as follows: s = singlet, d = doublet, dd = double doublet, m = multiplet; they are further described by their coupling constants *J*. Infrared spectra (IR) were measured as attenuated total reflection (ATR) experiments using a Nicolet 380 FT-IR spectrometer. The signals were characterised by their wavenumbers and corresponding absorption as very strong (vs), strong (s), medium (m), weak (w) or very weak (vw). UV–vis spectra were recorded on a Cary 60 UV–vis spectrophotometer, and emission spectra were recorded on an Agilent Cary Eclipse fluorescence spectrophotometer. Fluorescence quantum yields have been determined using the fluorescence standard quinine sulfate in 0.05 M H_2SO_4. Basic and high-resolution mass spectra (MS/HRMS) were measured on instruments coupled to a preceding gas chromatograph (GC) or liquid chromatograph (LC). Samples were ionised by electron impact ionisation (EI) on an Agilent 6890/5973 or Agilent 7890/5977 GC–MS equipped with an HP-5 capillary column using helium carrier gas or by electron spray ionisation (ESI) on an Agilent 1200/6210 Time-of-Flight (TOF) LC-MS. The solvents used, dimethylsulfoxide and toluene, were purchased as dry solvents and applied without further purification. Other reagents, catalysts, ligands, acids and bases were used as purchased from commercial suppliers. Column chromatography was performed on Merck Silica gel 60 (particle size 63–200 µm). Solvents for extraction and column chromatography were distilled prior employment. Compounds **2**, **3a**, **3b**, **3c**, **3d**, **3g**, **3h** were obtained as described previously and their characterisation data are in agreement with reported analysis [1]. Compounds **3e** and **3f** have been synthesized according to a known procedure [1].

Representative method for the preparation of starting materials

Representative method A for the synthesis of 4a–m.

A mixture of **3** (0.318 mmol; 102 mg), $Pd(OAc)_2$ (5 mol %; 0.027 mmol; 4 mg), DPEphos (5 mol %, 0.017 mmol, 9 mg) and K_3PO_4 (3 equiv, 0.993 mmol, 198 mg) were dissolved in DMA (5 ml) and stirred for 5 minutes. The corresponding aniline (1.1 equiv; 0.349 mmol, 37.4 mg) was added, and the reaction mixture was stirred at 100 °C for 15 hours. The reaction was neutralised with a saturated, aqueous NH_4Cl solution (1.0 M) and diluted with water (40 ml). The aqueous layer was extracted with dichloromethane (3 × 30 ml). The combined organic

layers were dried over Na_2SO_4, concentrated under reduced pressure, and purified by column chromatography (heptane/ethyl acetate).

5-Bromo-1,3-dimethyl-6-(*m*-tolylethynyl)pyrimidine-2,4(1*H*,3*H*)-dione (3e). According to the general procedure A of reference [1], compound **3e** was obtained as a brown solid in 97% yield (639 mg, 1.92 mmol, R_f = 0.37 heptane/ethyl acetate, 3:2); mp: 192–194 °C. IR (ATR): $\tilde{\nu}$ [cm^{-1}] = 1691 (s), 1650 (vs), 1537 (s), 1512 (s), 1432 (s), 1372 (m), 1211 (m), 824 (s). ^{1}H NMR (500 MHz, Chloroform-*d*) δ = 7.43 – 7.39 (m, 2H), 7.34 – 7.28 (m, 2H), 3.68 (s, 3H), 3.43 (s, 3H), 2.39 (s, 3H). ^{13}C {^{1}H} NMR (126 MHz, Chloroform-*d*) δ = 159.0, 151.0, 138.9, 136.3, 132.7, 132.0, 129.4, 128.8, 120.0, 107.1, 102.3, 80.4, 35.6, 29.6, 21.3. MS (EI, 70 eV): *m*/*z* (%) = 334 (100, M^{+}), 277 (29), 275 (14), 249 (17), 196 (31), 168 (63), 156 (94). HRMS (ESI-TOF): calcd. for $C_{15}H_{14}BrN_2O_2$ [M+H]$^{+}$ 333.0233, found: 333.0233.

5-Bromo-1,3-dimethyl-6-(*o*-tolylethynyl)pyrimidine-2,4(1*H*,3*H*)-dione (3f). According to the general procedure A of reference [1], compound **3f** was obtained as a brown solid in 60% yield (393 mg, 1.18 mmol, R_f = 0.37 heptane/ethyl acetate, 3:2); mp: 203–205 °C. IR (ATR): $\tilde{\nu}$ [cm^{-1}] = 2206 (m), 1706 (s), 1650 (vs), 1425 (s), 1246 (m), 1123 (m), 1005 (m), 762 (vs). ^{1}H NMR (500 MHz, Chloroform-*d*) δ = 7.57 (dd, *J* = 7.6, 1.4 Hz, 1H), 7.38 (ptd, *J* = 7.5, 1.4 Hz, 1H), 7.29 (d, *J* = 7.7 Hz, 1H), 7.24 (pt, *J* = 7.5 Hz, 1H), 3.69 (s, 3H), 3.43 (s, 3H), 2.54 (s, 3H). ^{13}C {^{1}H} NMR (126 MHz, Chloroform-*d*) δ = 159.0, 151.0, 141.7, 136.4, 133.0, 131.1, 130.2, 126.1, 120.2, 106.0, 102.0, 84.3, 35.5, 29.5, 21.1. MS (EI, 70 eV): *m*/*z* (%) = 332 (100, M^{+}), 275 (7), 196 (33), 168 (32), 157 (12), 140 (21), 115 (43). HRMS (ESI-TOF): calcd. For $C_{15}H_{14}BrN_2O_2$ [M+H]$^{+}$ 333.0233, found: 333.0242.

1,3-Dimethyl-5,6-di-*p*-tolyl-1,5-dihydro-2*H*-pyrrolo[3,2-*d*]pyrimidine-2,4(3*H*)-dione (4a). According to the representative method A, compound **4a** was obtained as a brown solid in 43% yield (45.8 mg, 127 µmol, R_f = 0.21 heptane/ethyl acetate, 3:2); mp: 250–252 °C. IR (ATR): $\tilde{\nu}$ [cm^{-1}] = 1687 (s), 1650 (vs), 1531 (s), 1512 (s), 1463 (s), 1444 (s), 1436 (s), 1399 (s). ^{1}H NMR (500 MHz, Chloroform-*d*) δ = 7.18 – 7.14 (m, 2H), 7.14 – 7.10 (m, 2H), 7.09 – 7.06 (m, 2H), 7.06 – 7.03 (m, 2H), 6.18 (s, 1H), 3.55 (s, 3H), 3.36 (s, 3H), 2.37 (s, 3H), 2.30 (s, 3H). ^{13}C {^{1}H} NMR (126 MHz, Chloroform-*d*) δ = 155.0, 151.8, 143.1, 138.4, 138.3, 136.5, 134.9, 129.3, 129.2, 128.9, 128.3, 127.9, 111.9, 95.2, 31.8, 28.0, 21.4, 21.3. MS (EI, 70 eV): *m*/*z* (%) = 359 (100, M^{+}), 301 (9), 273 (11), 259 (12), 227 (5), 180 (7), 115 (24). HRMS (ESI-TOF): calcd. for $C_{22}H_{22}N_3O_2$ [M+H]$^{+}$ 360.1712, found: 360.1719.

5-(4-Methoxyphenyl)-1,3-dimethyl-6-(*p*-tolyl)-1,5-dihydro-2*H*-pyrrolo[3,2-*d*]pyrimidine-2,4(3*H*)-dione (4b). According to the representative method A, compound **4b** was obtained as

a brown solid in 50% yield (56.3 mg, 150 µmol, R_f = 0.22 heptane/ethyl acetate, 3:2); mp: 212–214 °C. IR (ATR): $\tilde{\nu}$ [cm^{-1}] = 1693 (m), 1650 (m), 1644 (m), 1512 (m), 1055 (vs), 964 (s), 795 (s). ^{1}H NMR (300 MHz, Chloroform-*d*) δ = 7.17 – 7.13 (m, 2H), 7.09 – 7.03 (m, 4H), 6.89 – 6.85 (m, 2H), 6.17 (s, 1H), 3.81 (s, 3H), 3.55 (s, 3H), 3.36 (s, 3H), 2.30 (s, 3H). ^{13}C {^{1}H} NMR (75 MHz, Chloroform-*d*) δ = 159.4, 155.1, 151.8, 143.2, 138.5, 136.4, 130.3, 129.6, 129.2, 128.9, 127.9, 113.8, 112.0, 95.1, 55.5, 31.9, 28.0, 21.3. MS (EI, 70 eV): *m*/*z* (%) = 375 (71, M$^+$), 275 (5), 227 (7), 146 (96), 118 (56), 104 (47). HRMS (ESI-TOF): calcd. for $C_{22}H_{22}N_3O_3$ [M+H]$^+$ 376.1656, found: 376.1661.

1,3-Dimethyl-5-phenyl-6-(*p*-tolyl)-1,5-dihydro-2*H*-pyrrolo[3,2-*d*]pyrimidine-2,4(3*H*)-dione (4c). According to the representative method A, compound **4c** was obtained as a brown solid in 46% yield (47.3 mg, 137 µmol, R_f = 0.17 heptane/ethyl acetate, 3: 2); mp: 205–207 °C. IR (ATR): $\tilde{\nu}$ [cm^{-1}] = 1687 (s), 1650 (vs), 1531 (s), 1428 (s), 1312 (m), 1188 (m), 1020 (m), 768 (s). ^{1}H NMR (300 MHz, Chloroform-*d*) δ = 7.32 – 7.25 (m, 3H), 7.19 – 7.13 (m, 2H), 6.96 (m, 4H), 6.12 (s, 1H), 3.47 (s, 3H), 3.28 (s, 3H), 2.21 (s, 3H). ^{13}C {^{1}H} NMR (75 MHz, Chloroform-*d*) δ = 155.0, 151.8, 143.1, 138.5, 137.5, 136.6, 129.2, 128.9, 128.7, 128.6, 128.5, 127.8, 111.9, 95.4, 31.9, 28.0, 21.3. MS (EI, 70 eV): *m*/*z* (%) = 345 (100, M$^+$), 287 (13), 259 (23), 245 (5), 172 (7), 115 (26). HRMS (ESI-TOF): calcd. for $C_{21}H_{20}N_3O_2$ [M+H]$^+$ 346.1555, found: 346.1555.

1,3-Dimethyl-6-phenyl-5-(*p*-tolyl)-1,5-dihydro-2*H*-pyrrolo[3,2-*d*]pyrimidine-2,4(3*H*)-dione (4d). According to the representative method A, compound **4d** was obtained as a brown solid in 40% yield (43.9 mg, 127 µmol, R_f = 0.16 heptane/ethyl acetate, 3: 2); mp: 225–227 °C. IR (ATR): $\tilde{\nu}$ [cm^{-1}] = 1687 (s), 1650 (vs), 1512 (s), 1444 (s), 1374 (s), 1246 (m), 1116 (m), 1032 (m). ^{1}H NMR (300 MHz, Chloroform-*d*) δ = 7.20 – 7.14 (m, 3H), 7.14 – 7.02 (m, 6H), 6.13 (s, 1H), 3.48 (s, 3H), 3.29 (s, 3H), 2.29 (s, 3H). ^{13}C {^{1}H} NMR (75 MHz, Chloroform-*d*) δ = 155.1, 151.8, 142.9, 138.5, 136.5, 134.8, 130.9, 129.3, 129.1, 128.4, 128.4, 112.1, 95.5, 31.9, 28.0, 21.4 (signal of one carbon is absent, which may relate to signal overlap). MS (EI, 70 eV): *m*/*z* (%) = 345 (100, M$^+$), 293 (44), 287 (71), 273 (19), 259 (84), 245 (74), 228 (27). HRMS (ESI-TOF): calcd. for $C_{21}H_{20}N_3O_2$ [M+H]$^+$ 346.1555, found: 346.1561.

1,3-Dimethyl-5-(*o*-tolyl)-6-(*p*-tolyl)-1,5-dihydro-2*H*-pyrrolo[3,2-*d*]pyrimidine-2,4(3*H*)-dione (4e). According to the representative method A, compound **4e** was obtained as a brown solid in 53% yield (108 mg, 301 µmol, R_f = 0.29 heptane/ethyl acetate, 3:2); mp: 166–168 °C. IR (ATR): $\tilde{\nu}$ [cm^{-1}] = 1693 (s), 1650 (vs), 1531 (m), 1425 (m), 1327 (m), 1242 (m), 1127 (m), 745 (s). ^{1}H NMR (500 MHz, Chloroform-*d*) δ = 7.35 – 7.29 (m, 1H), 7.25 – 7.20 (m, 3H), 7.09 – 7.05 (m, 2H), 7.05 – 7.00 (m, 2H), 6.23 (s, 1H), 3.57 (s, 3H), 3.35 (s, 3H), 2.28 (s, 3H),

1.96 (s, 3H). ^{13}C {^{1}H} NMR (126 MHz, Chloroform-*d*) δ = 155.0, 151.9, 142.9, 138.6, 137.1, 136.5, 136.4, 130.7, 129.2, 129.1, 129.1, 128.4, 127.8, 126.4, 111.7, 94.7, 31.9, 28.0, 21.3, 17.7. MS (EI, 70 eV): *m*/*z* (%) = 359 (21, M^+), 344 (86), 341 (45), 301 (63), 273 (89), 259 (80), 245 (60). HRMS (ESI-TOF): calcd. for $C_{22}H_{21}N_3O_2$ $[M+H]^+$ 360.1707, found: 360.1711.

5-(2-Bromophenyl)-1,3-dimethyl-6-(*p*-tolyl)-1,5-dihydro-2*H*-pyrrolo[3,2-*d*]pyrimidine-2,4(3*H*)-dione (4f). According to the representative method A, compound **4f** was obtained as a brown solid in 39% yield (51 mg, 120 µmol, R_f = 0.25 heptane/ethyl acetate, 3:2); mp: 157–159 °C. IR (ATR): $\tilde{\nu}$ [cm^{-1}] = 1691 (s), 1648 (vs), 1477 (s), 1318 (m), 1195 (m), 1028 (m), 797 (s), 745 (s). ^{1}H NMR (300 MHz, Chloroform-*d*) δ = 7.64 – 7.60 (m, 1H), 7.35 – 7.24 (m, 3H), 7.14 – 7.10 (m, 2H), 7.06 – 7.02 (m, 2H), 6.23 (s, 1H), 3.56 (s, 3H), 3.35 (s, 3H), 2.29 (s, 3H). ^{13}C {^{1}H} NMR (75 MHz, Chloroform-*d*) δ = 154.9, 151.9, 143.2, 138.9, 137.4, 136.5, 133.1, 130.8, 130.5, 129.3, 128.7, 127.9, 127.5, 124.1, 111.8, 95.4, 32.0, 28.0, 21.4. MS (EI, 70 eV): *m*/*z* (%) = 423(79, M^+), 345 (64), 328 (12), 287 (24), 259 (18), 231 (16), 216 (10). HRMS (ESI-TOF): calcd. for $C_{21}H_{19}BrN_3O_2$ $[M+H]^+$ 424.0655, found: 424.0664.

1,3-Dimethyl-6-(*o*-tolyl)-5-(*p*-tolyl)-1,5-dihydro-2*H*-pyrrolo[3,2-*d*]pyrimidine-2,4(3*H*)-dione (4g). According to the representative method A, compound **4g** was obtained as a brown solid in 48% yield (78 mg, 217 µmol, R_f = 0.27 heptane/ethyl acetate, 3:2); mp: 230–232 °C. IR (ATR): $\tilde{\nu}$ [cm^{-1}] = 1693 (vs), 1650 (vs), 1514 (s), 1403 (m), 1333 (m), 1240 (m), 1106 (m), 762 (s). ^{1}H NMR (300 MHz, Chloroform-*d*) δ = 7.24 – 7.18 (m, 1H), 7.14 – 7.08 (m, 3H), 7.07 – 6.99 (m, 4H), 6.07 (s, 1H), 3.55 (s, 3H), 3.38 (s, 3H), 2.29 (s, 3H), 2.10 (s, 3H). ^{13}C {^{1}H} NMR (75 MHz, Chloroform-*d*) δ = 155.1, 151.9, 142.8, 137.9, 137.6, 136.4, 134.3, 131.3, 130.7, 130.2, 129.1, 128.9, 127.7, 125.5, 111.0, 96.4, 31.9, 28.0, 21.3, 20.3. MS (EI, 70 eV): *m*/*z* (%) = 359 (100, M^+), 344 (10), 273 (8), 259 (7), 115 (15). HRMS (EI): calcd. for $C_{22}H_{21}N_3O_2$ $[M]^+$ 359.16283, found: 359.16196.

1,3-Dimethyl-6-(*m*-tolyl)-5-(*p*-tolyl)-1,5-dihydro-2*H*-pyrrolo[3,2-*d*]pyrimidine-2,4(3*H*)-dione (4h). According to the representative method A, compound **4h** was obtained as a brown solid in 26% yield (42.1 mg, 117 µmol, R_f = 0.28 heptane/ethyl acetate, 3: 2); mp: 211–213 °C. IR (ATR): $\tilde{\nu}$ [cm^{-1}] = 1691 (vs), 1654 (vs), 1531 (s), 1475 (s), 1372 (s), 1207 (m), 110 (m), 748 (s). ^{1}H NMR (300 MHz, Chloroform-*d*) δ = 7.18 – 7.04 (m, 7H), 6.94 – 6.88 (m, 1H), 6.20 (s, 1H), 3.55 (s, 3H), 3.36 (s, 3H), 2.37 (s, 3H), 2.26 (s, 3H). ^{13}C {^{1}H} NMR (75 MHz, Chloroform-*d*) δ = 155.1, 151.8, 143.1, 138.4, 138.1, 136.4, 134.9, 130.7, 129.8, 129.3, 129.2, 128.3, 128.2, 126.2, 112.1, 95.4, 31.9, 28.0, 21.4, 21.4 (signals of two carbons are absent, which may relate to signal overlap). MS (EI, 70 eV): *m*/*z* (%) = 359 (100, M^+), 301 (22), 273 (31), 259 (31), 242 (8), 129 (11). HRMS (EI): calcd. for $C_{22}H_{21}N_3O_2$ $[M]^+$ 359.16283, found: 359.16188.

5-(4-Fluorophenyl)-1,3-dimethyl-6-(*p*-tolyl)-1,5-dihydro-2*H*-pyrrolo[3,2-*d*]pyrimidine-2,4(3*H*)-dione (4i). According to the representative method A, compound **4i** was obtained as a brown solid in 42% yield (45.8 mg, 126 µmol, R_f = 0.17 heptane/ethyl acetate, 3: 2); mp: 208–210 °C. IR (ATR): $\tilde{\nu}$ [cm^{-1}] = 1687 (s), 1654 (vs), 1531 (s), 1506 (s), 1417 (s), 1463 (s), 1224 (s), 805 (vs). ^{1}H NMR (300 MHz, Chloroform-*d*) δ = 7.24 – 7.18 (m, 2H), 7.04 (m, 6H), 6.19 (s, 1H), 3.55 (s, 3H), 3.36 (s, 3H), 2.31 (s, 3H). ^{19}F NMR (282 MHz, Chloroform-*d*) δ = -112.7. ^{13}C {^{1}H} NMR (75 MHz, Chloroform-*d*) δ = 162.2 (d, J = 248.4 Hz), 155.1, 151.7, 143.3, 138.8, 136.6, 133.5 (d, J = 3.3 Hz), 130.4 (d, J = 8.8 Hz), 129.3, 129.0, 127.6, 115.6 (d, J = 23.0 Hz), 111.9, 95.6, 31.9, 28.0, 21.3. MS (EI, 70 eV): *m*/*z* (%) = 363 (100, M$^+$), 305 (19), 277 (44), 227 (9), 182 (11), 142 (8). HRMS (ESI-TOF): calcd. for $C_{21}H_{19}FN_3O_2$ [M+H]$^+$ 364.1461, found: 364.1462.

1,3-Dimethyl-6-(*p*-tolyl)-5-(4-(trifluoromethyl)phenyl)-1,5-dihydro-2*H*-pyrrolo[3,2-*d*]-pyrimidine-2,4(3*H*)-dione (4j). According to the representative method A, compound **4j** was obtained as a brown solid in 38% yield (48 mg, 116 µmol, R_f = 0.19 heptane/ethyl acetate, 3:2); mp: 250–252 °C. IR (ATR): $\tilde{\nu}$ [cm^{-1}] = 1687 (s), 1654 (vs), 1528 (s), 1333 (vs), 1156 (s), 1110 (vs), 1069 (s), 984 (s). ^{1}H NMR (300 MHz, Chloroform-*d*) δ = 7.63 – 7.59 (m, 2H), 7.39 – 7.33 (m, 2H), 7.08 – 6.98 (m, 4H), 6.23 (s, 1H), 3.56 (s, 3H), 3.36 (s, 3H), 2.31 (s, 3H). ^{19}F NMR (282 MHz, Chloroform-*d*) δ = -62.5. ^{13}C {^{1}H} NMR (75 MHz, Chloroform-*d*) δ = 155.1, 151.7, 143.3, 140.4, 139.0, 137.0, 130.3 (q, J = 33.0 Hz), 129.4, 129.3, 129.2, 129.0, 127.3, 125.7 (q, J = 3.7 Hz), 122.1, 118.5, 111.6, 96.3, 32.0, 28.1, 21.3 (signals of five carbons are absent, which may relate to signal overlap). MS (EI, 70 eV): *m*/*z* (%) = 413 (100, M$^+$), 327 (11), 259 (4), 205 (5). HRMS (ESI-TOF): calcd. for $C_{22}H_{19}F_3N_3O_2$ [M+H]$^+$ 414.1429, found: 414.1437.

6-(4-(Dimethylamino)phenyl)-1,3-dimethyl-5-(*p*-tolyl)-1,5-dihydro-2*H*-pyrrolo[3,2-*d*]pyrimidine-2,4(3*H*)-dione (4k). According to the representative method A, compound **4k** was obtained as a brown solid in 33% yield (35.6 mg, 91.6 µmol, R_f = 0.13 heptane/ethyl acetate, 3:2); mp: 209–211 °C. IR (ATR): $\tilde{\nu}$ [cm^{-1}] = 1681 (s), 1652 (vs), 1605 (s), 1514 (s), 1432 (s), 1355 (s), 1246 (s), 1120 (s). ^{1}H NMR (300 MHz, Chloroform-*d*) δ = 7.16 (d, J = 1.9 Hz, 4H), 7.07 – 7.00 (m, 2H), 6.57 – 6.51 (m, 2H), 6.10 (s, 1H), 3.54 (s, 3H), 3.35 (s, 3H), 2.93 (s, 6H), 2.38 (s, 3H). ^{13}C {^{1}H} NMR (75 MHz, Chloroform-*d*) δ = 154.9, 151.9, 150.2, 143.9, 138.2, 136.7, 135.4, 129.9, 129.3, 128.5, 118.2, 111.7, 111.5, 94.0, 40.2, 31.9, 28.0, 21.4. MS (EI, 70 eV): *m*/*z* (%) = 388 (100, M$^+$), 274 (29), 366 (87), 331 (16), 302 (31), 288 (29), 270 (79). HRMS (ESI-TOF): calcd. for $C_{23}H_{25}N_4O_2$ [M+H]$^+$ 389.1977, found: 389.1980.

6-(4-(Dimethylamino)phenyl)-1,3-dimethyl-5-(4-(zuoromethyl)phenyl)-1,5-dihydro-2*H*-pyrrolo[3,2-*d*]pyrimidine-2,4(3*H*)-dione (4l). According to the representative method A, compound **4l** was obtained as a brown solid in 23% yield (28.6 mg, 64.6 µmol, R_f = 0.23 heptane/ethyl acetate, 3:2); mp: 269–271 °C. IR (ATR): $\tilde{\nu}$ [cm^{-1}] = 1683 (s), 1652 (vs), 1603 (s), 1473 (s), 1397 (s), 1329 (vs), 1156 (vs), 1110 (vs). ^{1}H NMR (300 MHz, Chloroform-*d*) δ = 7.66 – 7.59 (m, 2H), 7.42 – 7.36 (m, 2H), 7.00 – 6.94 (m, 2H), 6.57 – 6.51 (m, 2H), 6.15 (s, 1H), 3.55 (s, 3H), 3.36 (s, 3H), 2.95 (s, 6H). ^{19}F NMR (282 MHz, Chloroform-*d*) δ = -62.4. ^{13}C {^{1}H} NMR (75 MHz, Chloroform-*d*) δ = 155.0, 151.7, 150.4, 144.2, 140.9, 137.3, 130.1 (q, *J* = 32.8 Hz), 130.0, 129.3, 125.7 (q, *J* = 3.8 Hz), 124.0 (d, *J* = 272.4 Hz), 117.3, 111.8, 111.1, 95.1, 40.2, 32.0, 28.0. MS (EI, 70 eV): *m*/*z* (%) = 442 (41, M$^+$), 428 (55), 423 (21), 384 (15), 356 (28), 340 (9), 270 (31). HRMS (ESI-TOF): calcd. for $C_{23}H_{21}F_3N_4O_2$ [M+H]$^+$ 443.1689, found: 443.1694.

1,3-Dimethyl-6-(thiophen-3-yl)-5-(*p*-tolyl)-1,5-dihydro-2*H*-pyrrolo[3,2-*d*]pyrimidine-2,4(3*H*)-dione (4m). According to the representative method A, compound **4m** was obtained as a brown solid in 39% yield (42.9 mg, 122 µmol, R_f = 0.10 heptane/ethyl acetate, 3:2); mp: 208–210 °C. IR (ATR): $\tilde{\nu}$ [cm^{-1}] = 1687, 1648 (vs), 1533 (s), 1514 (s), 1413 (s), 1287 (m), 1114 (m), 966 (m), 778 (s). ^{1}H NMR (300 MHz, Chloroform-*d*) δ = 7.2 – 7.1 (m, 5H), 6.8 (dd, *J* = 5.1, 1.3 Hz, 1H), 6.8 (dd, *J* = 2.9, 1.3 Hz, 1H), 6.2 (s, 1H), 3.5 (s, 3H), 3.3 (s, 3H), 2.4 (s, 3H). ^{13}C {^{1}H} NMR (75 MHz, Chloroform-*d*) δ = 155.0, 151.8, 139.1, 138.1, 136.3, 135.2, 131.3, 129.7, 128.2, 127.5, 125.7, 124.0, 112.0, 94.6, 31.9, 28.0, 21.5. MS (EI, 70 eV): *m*/*z* (%) = 351 (100, M$^+$), 336 (19), 293 (83), 279 (30), 265 (87), 251 (83), 237 (26). HRMS (ESI-TOF): calcd. for $C_{19}H_{18}N_3O_2S$ [M+H]$^+$ 352.1120, found: 352.1127.

^{1}H, ^{13}C, ^{19}F NMR spectra
5-Bromo-1,3-dimethyl-6-(*m*-tolylethynyl)pyrimidine-2,4(1*H*,3*H*)-dione (3e)

	Parameter	Value
1	Solvent	CDCl3
2	Temperature	298.1
3	Pulse Sequence	zg30
4	Experiment	1D
5	Number of Scans	16
6	Receiver Gain	101.0
7	Relaxation Delay	1.0000
8	Pulse Width	8.0000
9	Spectrometer Frequency	500.13
10	Spectral Width	10000.0
11	Lowest Frequency	-1958.2
12	Nucleus	1H
13	Acquired Size	32768
14	Spectral Size	65536

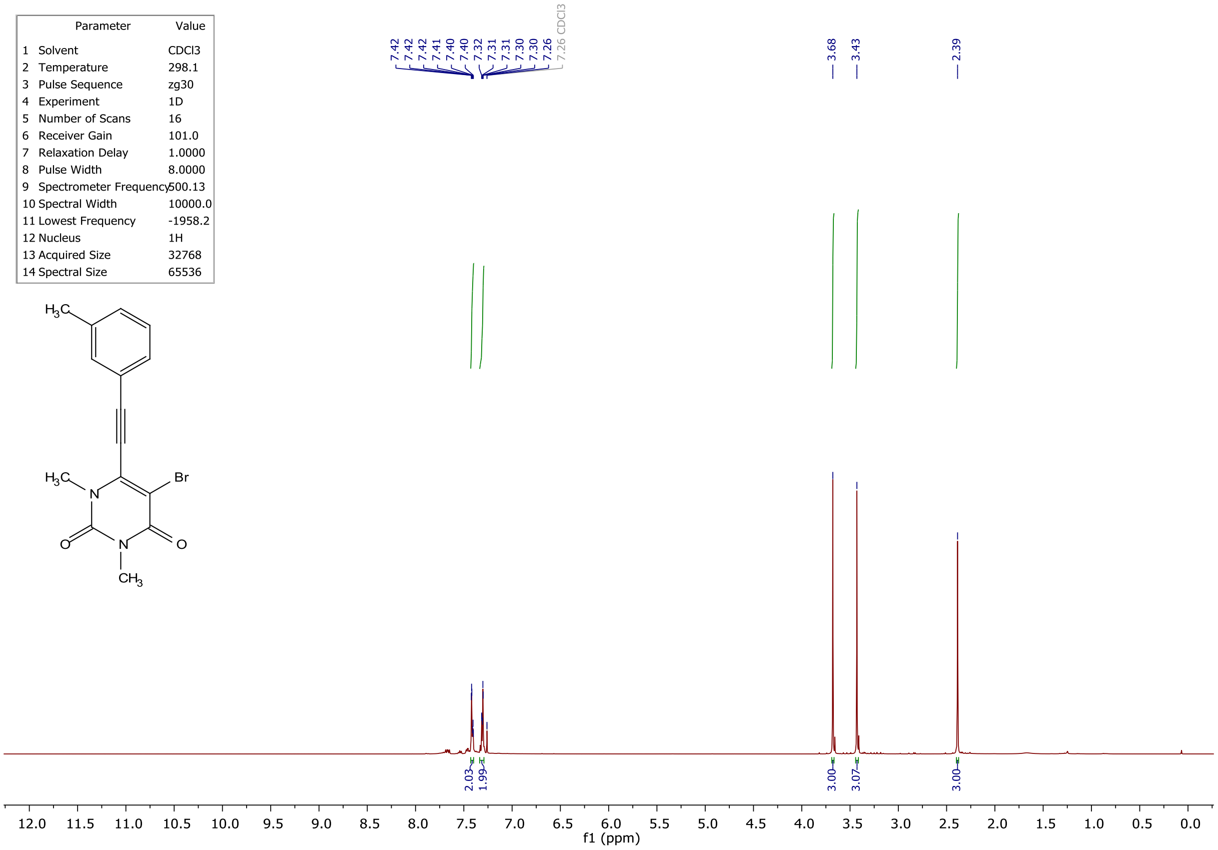

	Parameter	Value
1	Solvent	CDCl3
2	Temperature	298.2
3	Pulse Sequence	zgpg30
4	Experiment	1D
5	Number of Scans	1024
6	Receiver Gain	101.0
7	Relaxation Delay	2.0000
8	Pulse Width	10.0000
9	Spectrometer Frequency	125.77
10	Spectral Width	30120.5
11	Lowest Frequency	-2471.5
12	Nucleus	13C
13	Acquired Size	32768
14	Spectral Size	65536

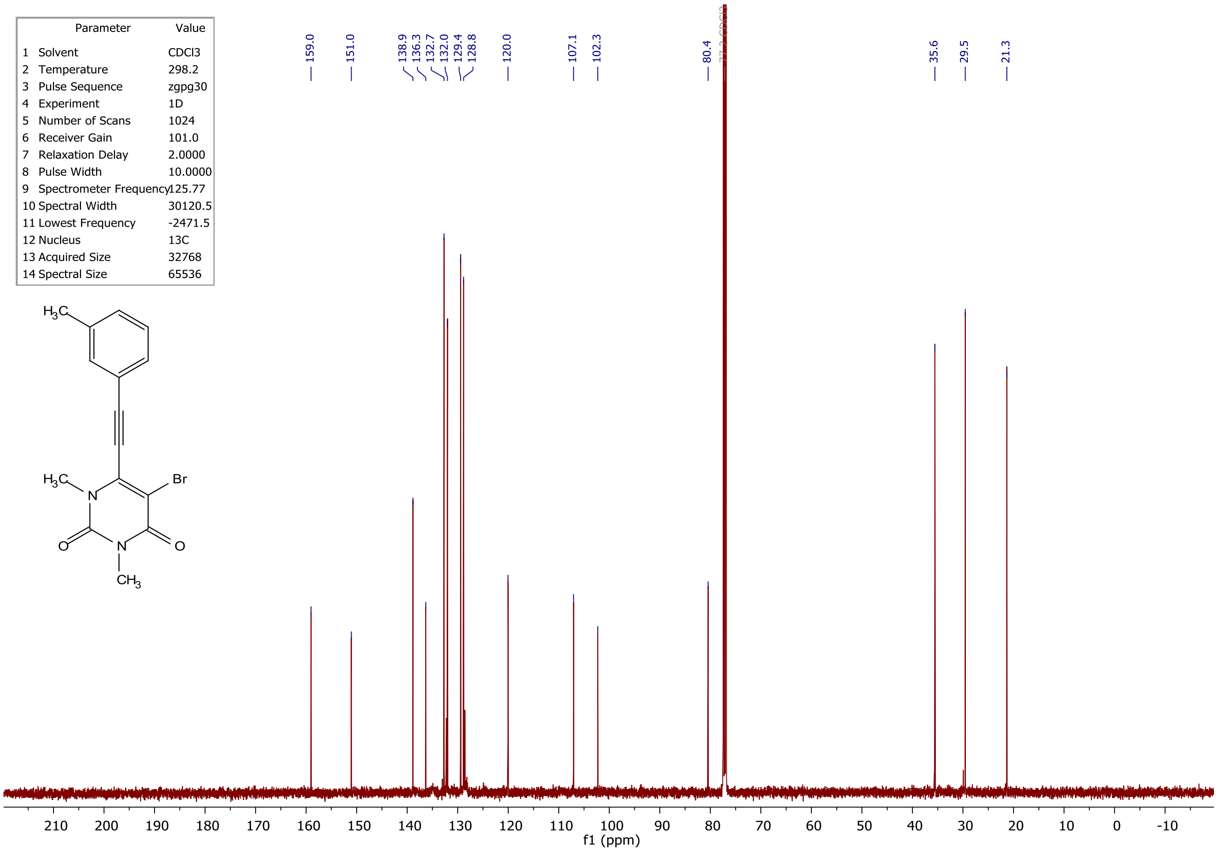

5-Bromo-1,3-dimethyl-6-(*o*-tolylethynyl)pyrimidine-2,4(1*H*,3*H*)-dione (3f)

	Parameter	Value
1	Solvent	CDCl3
2	Temperature	298.2
3	Pulse Sequence	zg30
4	Experiment	1D
5	Number of Scans	16
6	Receiver Gain	101.0
7	Relaxation Delay	1.0000
8	Pulse Width	8.0000
9	Spectrometer Frequency	500.13
10	Spectral Width	10000.0
11	Lowest Frequency	-1924.8
12	Nucleus	1H
13	Acquired Size	32768
14	Spectral Size	65536

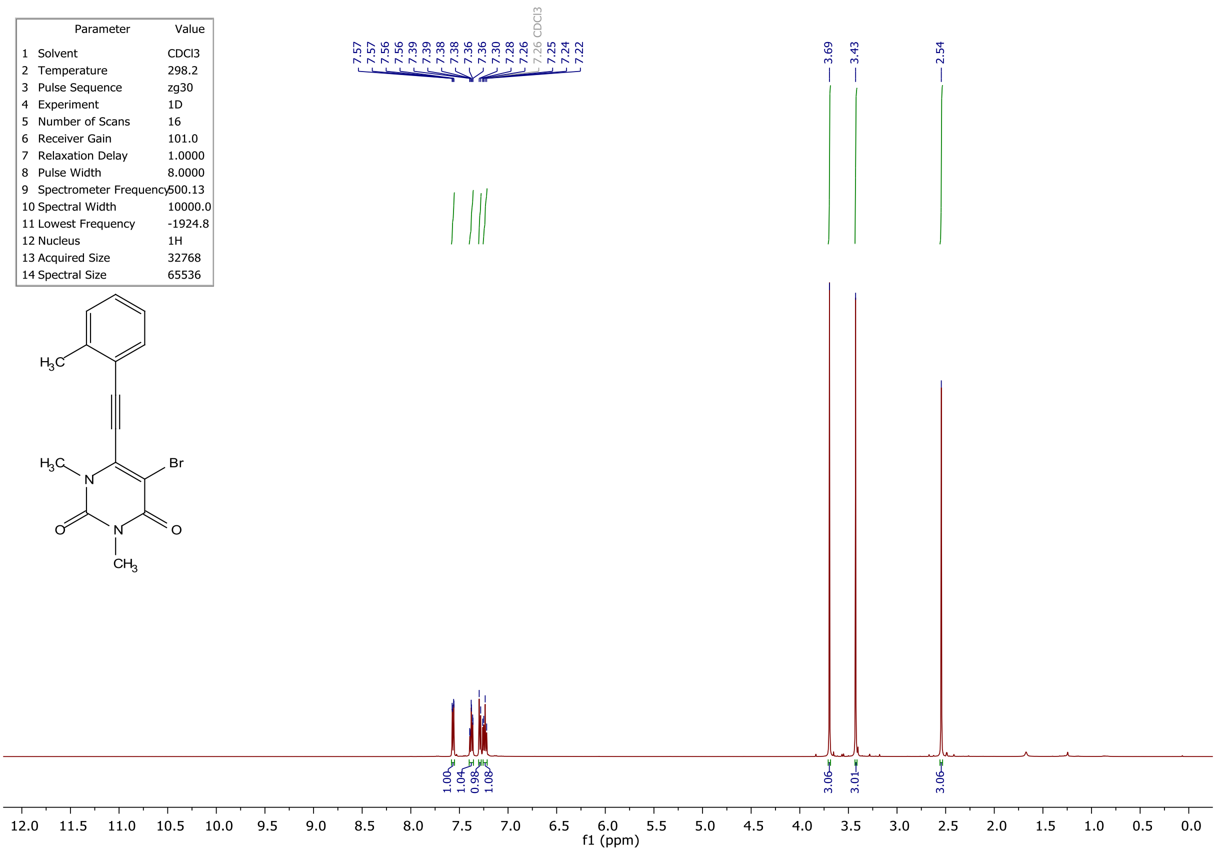

	Parameter	Value
1	Solvent	CDCl3
2	Temperature	298.2
3	Pulse Sequence	zgpg30
4	Experiment	1D
5	Number of Scans	1024
6	Receiver Gain	101.0
7	Relaxation Delay	2.0000
8	Pulse Width	10.0000
9	Spectrometer Frequency	125.77
10	Spectral Width	30120.5
11	Lowest Frequency	-2472.8
12	Nucleus	13C
13	Acquired Size	32768
14	Spectral Size	65536

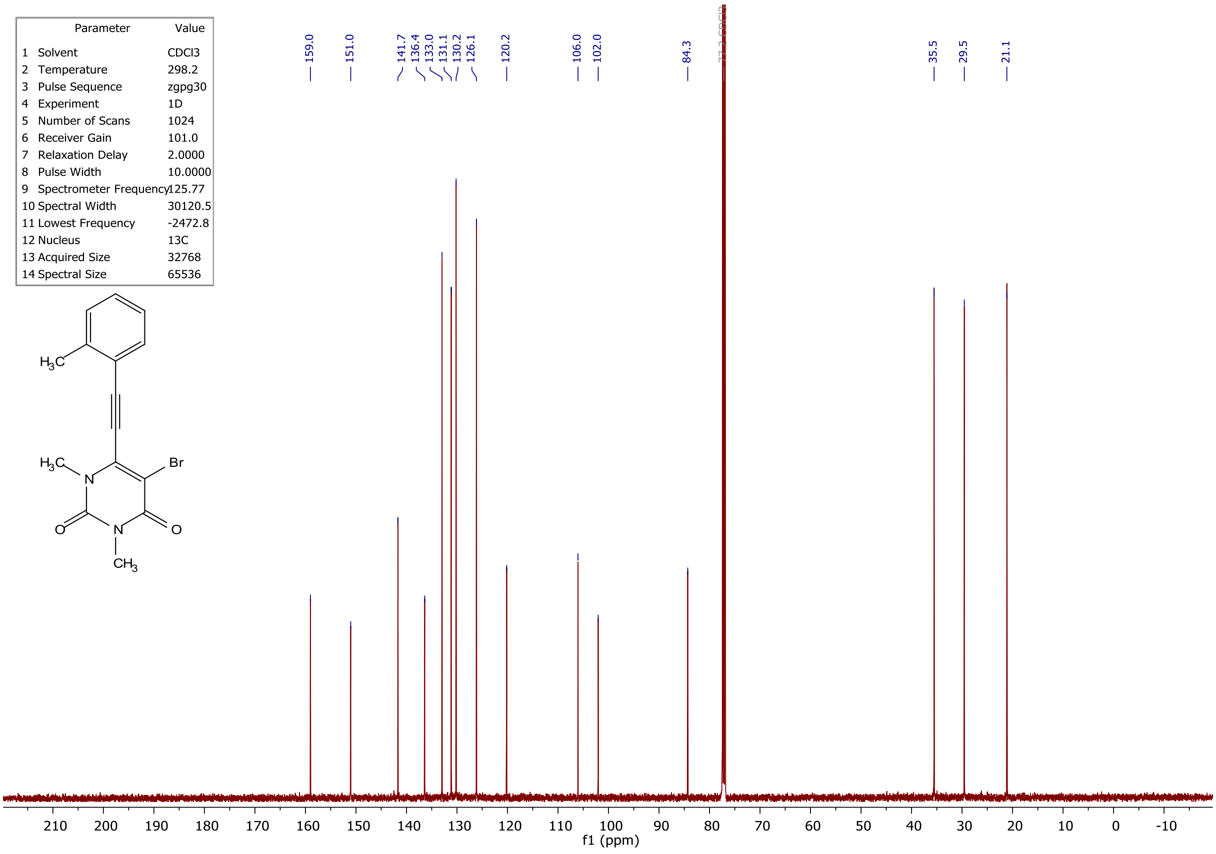

1,3-Dimethyl-5,6-di-*p*-tolyl-1,5-dihydro-2*H*-pyrrolo[3,2-*d*]pyrimidine-2,4(3*H*)-dione (4a).

	Parameter	Value
1	Solvent	CDCl3
2	Temperature	298.2
3	Pulse Sequence	zg30
4	Experiment	1D
5	Number of Scans	16
6	Receiver Gain	71.8
7	Relaxation Delay	1.0000
8	Pulse Width	8.0000
9	Spectrometer Frequency	500.13
10	Spectral Width	10000.0
11	Lowest Frequency	-1924.4
12	Nucleus	1H
13	Acquired Size	32768
14	Spectral Size	65536

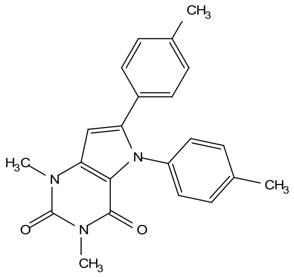

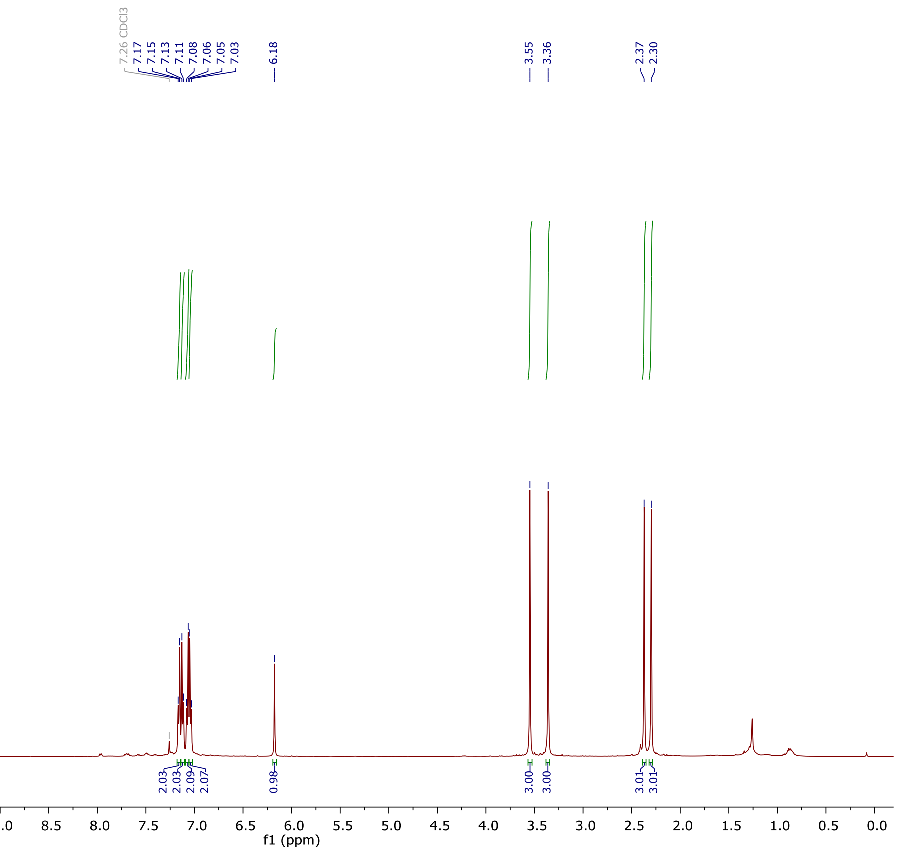

	Parameter	Value
1	Solvent	CDCl3
2	Temperature	298.2
3	Pulse Sequence	zgpg30
4	Experiment	1D
5	Number of Scans	1024
6	Receiver Gain	101.0
7	Relaxation Delay	2.0000
8	Pulse Width	10.0000
9	Spectrometer Frequency	125.77
10	Spectral Width	30120.5
11	Lowest Frequency	-2476.7
12	Nucleus	13C
13	Acquired Size	32768
14	Spectral Size	65536

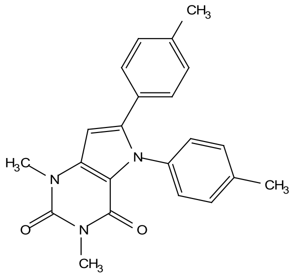

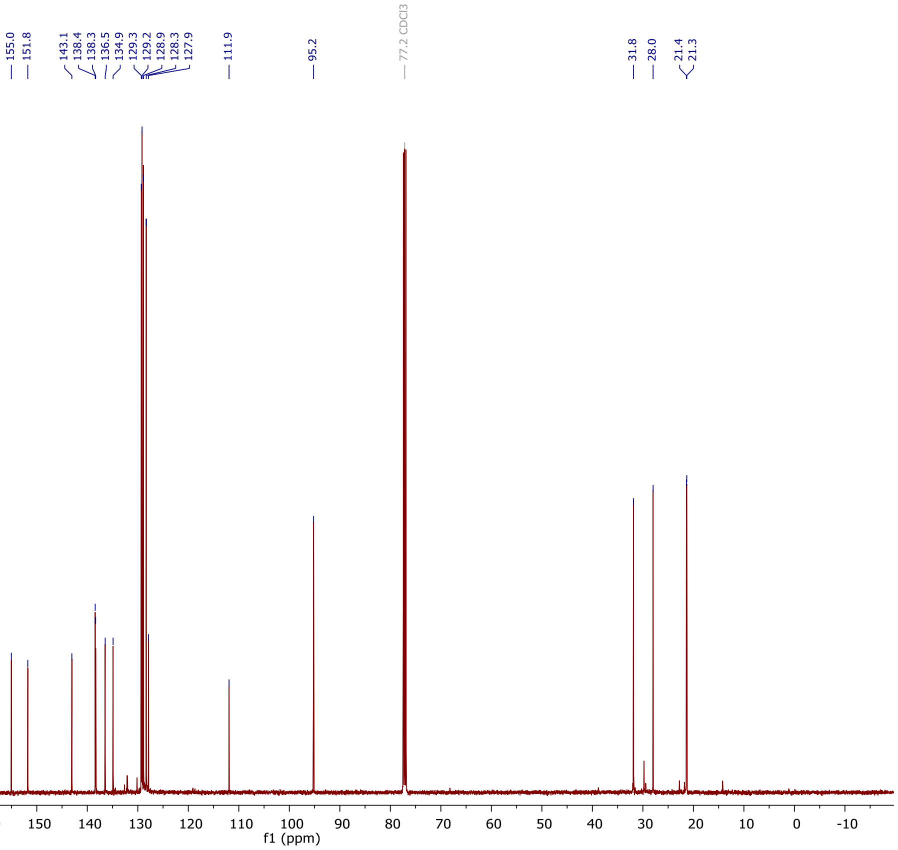

5-(4-Methoxyphenyl)-1,3-dimethyl-6-(*p*-tolyl)-1,5-dihydro-2*H*-pyrrolo[3,2-*d*]pyrimidine-2,4(3*H*)-dione (4b)

	Parameter	Value
1	Solvent	CDCl3
2	Temperature	298.1
3	Pulse Sequence	zg30
4	Experiment	1D
5	Number of Scans	16
6	Receiver Gain	101.0
7	Relaxation Delay	1.0000
8	Pulse Width	8.0000
9	Spectrometer Frequency	500.13
10	Spectral Width	10000.0
11	Lowest Frequency	-1924.8
12	Nucleus	1H
13	Acquired Size	32768
14	Spectral Size	65536

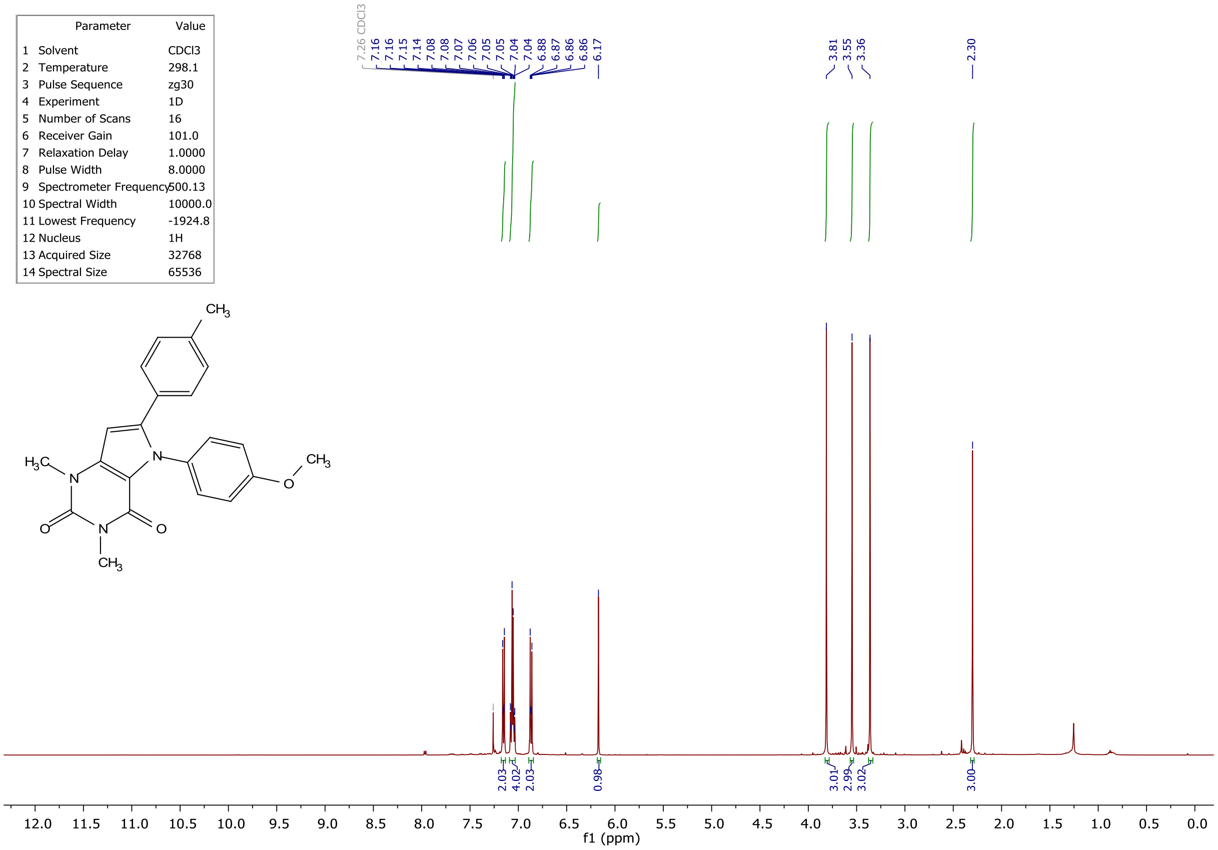

	Parameter	Value
1	Solvent	CDCl3
2	Temperature	298.2
3	Pulse Sequence	zgpg30
4	Experiment	1D
5	Number of Scans	1024
6	Receiver Gain	101.0
7	Relaxation Delay	2.0000
8	Pulse Width	10.0000
9	Spectrometer Frequency	125.77
10	Spectral Width	30120.5
11	Lowest Frequency	-2472.9
12	Nucleus	13C
13	Acquired Size	32768
14	Spectral Size	65536

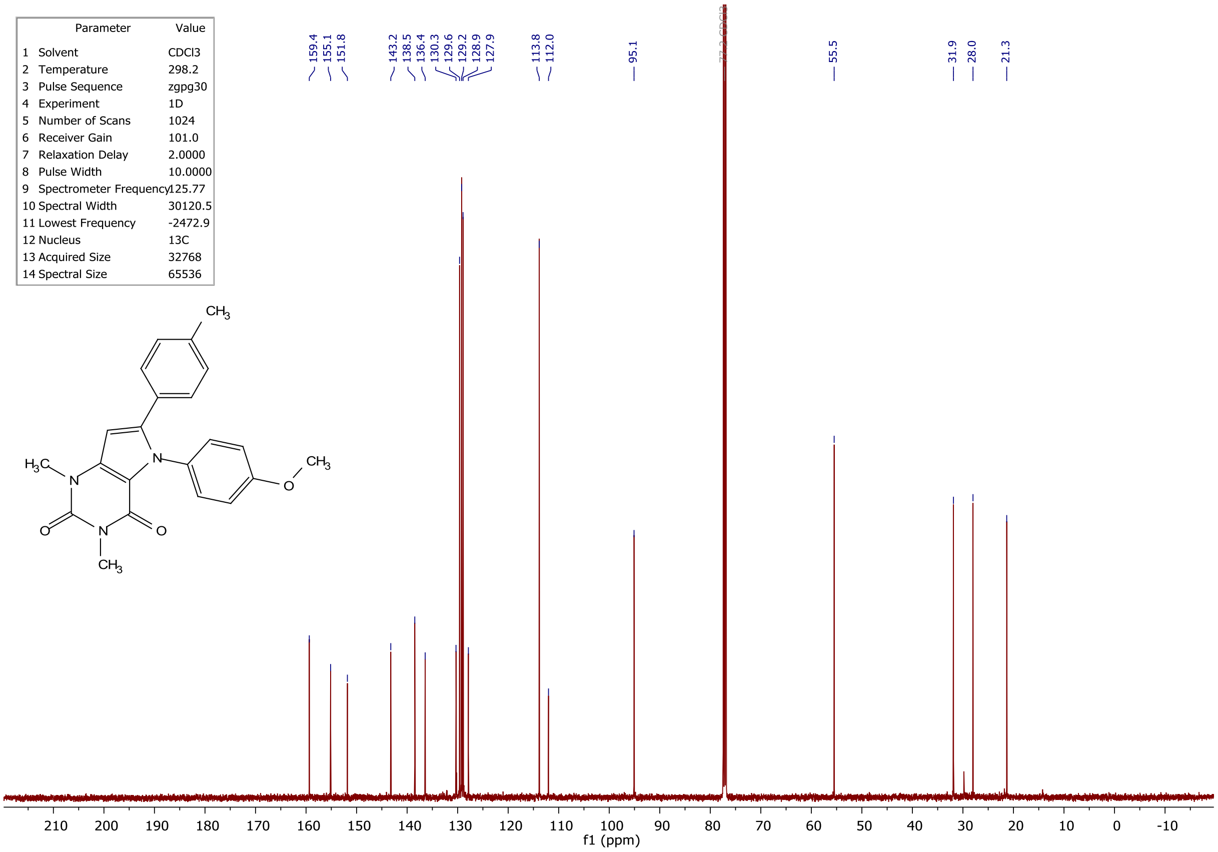

1,3-Dimethyl-5-phenyl-6-(*p*-tolyl)-1,5-dihydro-2*H*-pyrrolo[3,2-*d*]pyrimidine-2,4(3*H*)-dione (4c)

	Parameter	Value
1	Solvent	CDCl3
2	Temperature	298.2
3	Pulse Sequence	zg30
4	Experiment	1D
5	Number of Scans	16
6	Receiver Gain	71.8
7	Relaxation Delay	2.0000
8	Pulse Width	10.0000
9	Spectrometer Frequency	300.13
10	Spectral Width	6188.1
11	Lowest Frequency	-1269.9
12	Nucleus	1H
13	Acquired Size	32768
14	Spectral Size	65536

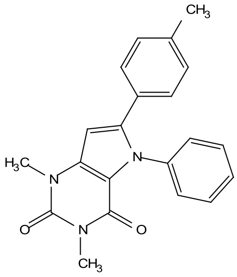

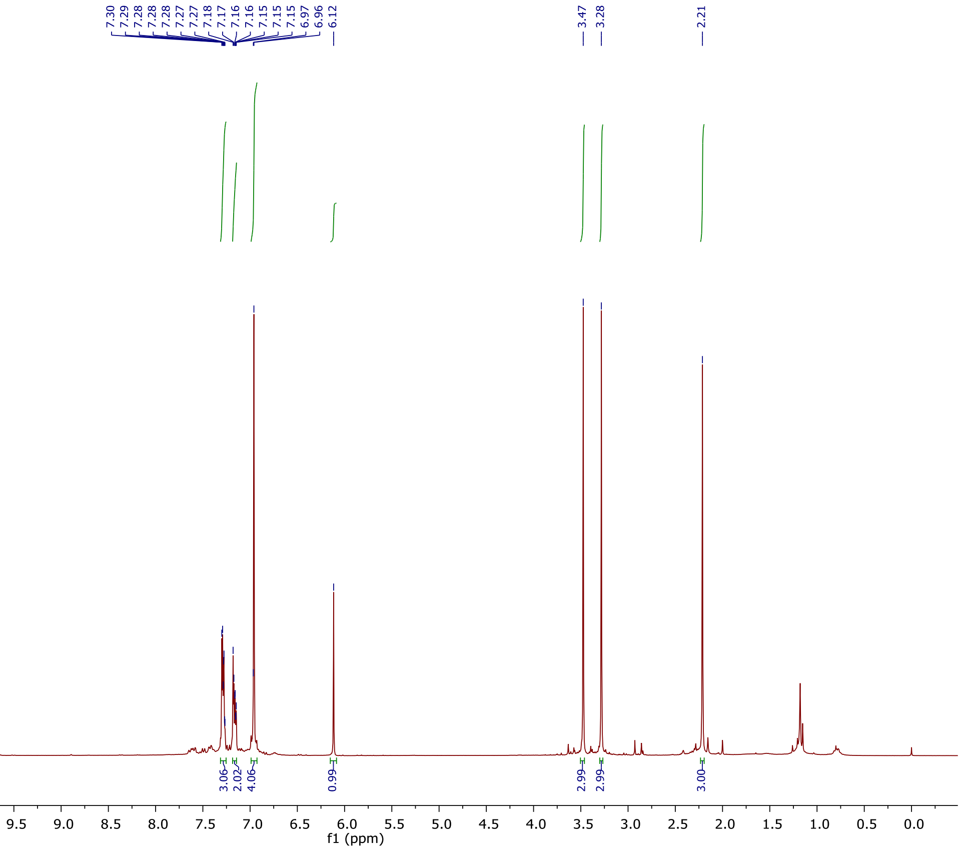

	Parameter	Value
1	Solvent	CDCl3
2	Temperature	299.8
3	Pulse Sequence	zgpg30
4	Experiment	1D
5	Number of Scans	1024
6	Receiver Gain	2050.0
7	Relaxation Delay	2.0000
8	Pulse Width	10.0000
9	Spectrometer Frequency	75.48
10	Spectral Width	18028.8
11	Lowest Frequency	-1461.6
12	Nucleus	13C
13	Acquired Size	32768
14	Spectral Size	65536

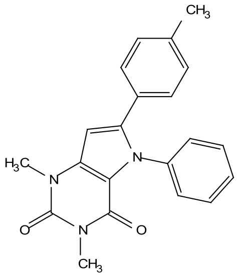

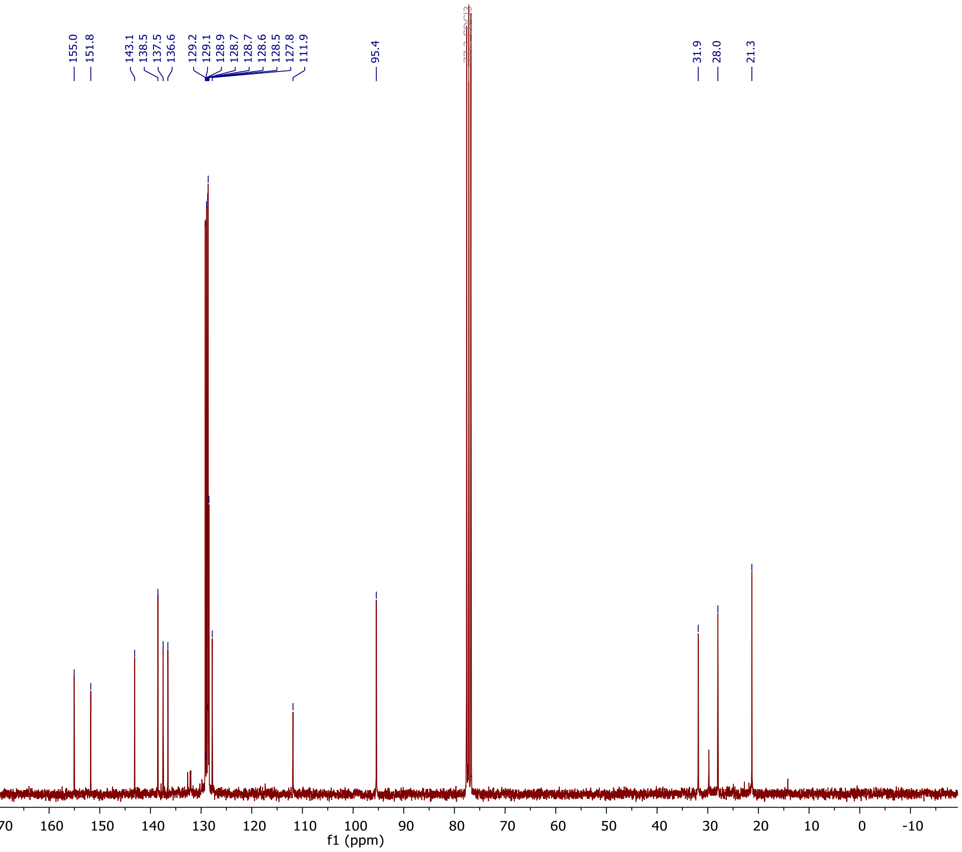

1,3-Dimethyl-6-phenyl-5-(*p*-tolyl)-1,5-dihydro-2*H*-pyrrolo[3,2-*d*]pyrimidine-2,4(3*H*)-dione (4d)

Parameter	Value
1 Solvent	CDCl3
2 Temperature	298.6
3 Pulse Sequence	zg30
4 Experiment	1D
5 Number of Scans	8
6 Receiver Gain	203.0
7 Relaxation Delay	2.0000
8 Pulse Width	10.0000
9 Spectrometer Frequency	300.13
10 Spectral Width	6188.1
11 Lowest Frequency	-1269.0
12 Nucleus	1H
13 Acquired Size	32768
14 Spectral Size	65536

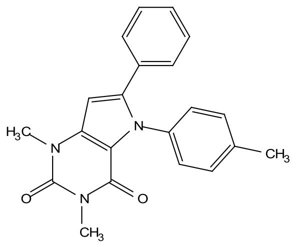

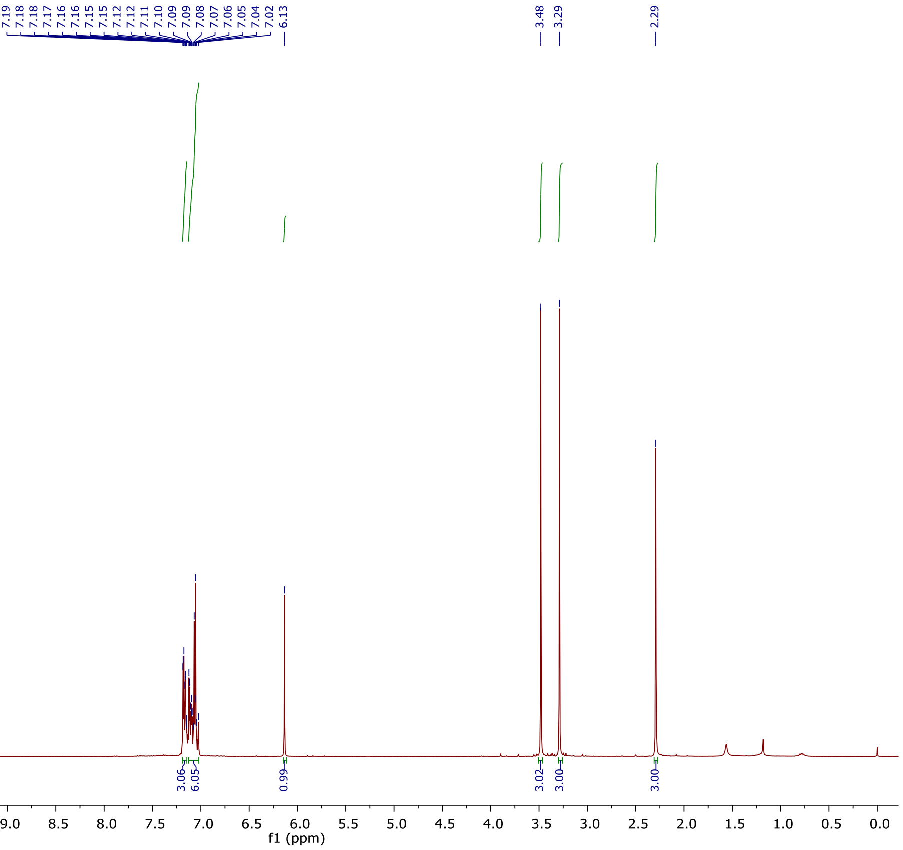

Parameter	Value
1 Solvent	CDCl3
2 Temperature	299.8
3 Pulse Sequence	zgpg30
4 Experiment	1D
5 Number of Scans	1024
6 Receiver Gain	2050.0
7 Relaxation Delay	2.0000
8 Pulse Width	10.0000
9 Spectrometer Frequency	75.48
10 Spectral Width	18028.8
11 Lowest Frequency	-1458.9
12 Nucleus	13C
13 Acquired Size	32768
14 Spectral Size	65536

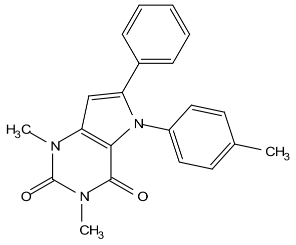

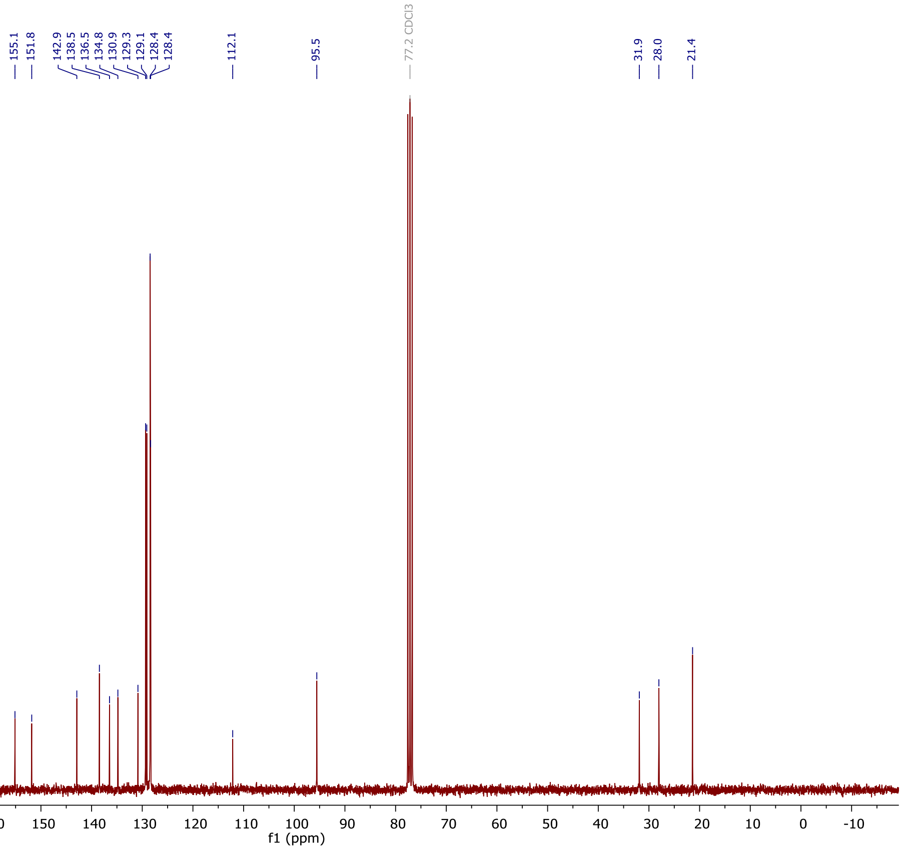

1,3-Dimethyl-5-(*o*-tolyl)-6-(*p*-tolyl)-1,5-dihydro-2*H*-pyrrolo[3,2-*d*]pyrimidine-2,4(3*H*)-dione (4e)

	Parameter	Value
1	Solvent	CDCl3
2	Temperature	298.2
3	Pulse Sequence	zg30
4	Experiment	1D
5	Number of Scans	16
6	Receiver Gain	101.0
7	Relaxation Delay	1.0000
8	Pulse Width	8.0000
9	Spectrometer Frequency	500.13
10	Spectral Width	10000.0
11	Lowest Frequency	-1924.8
12	Nucleus	1H
13	Acquired Size	32768
14	Spectral Size	65536

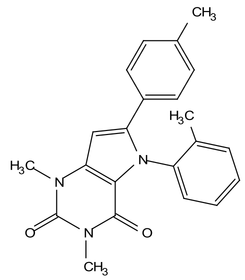

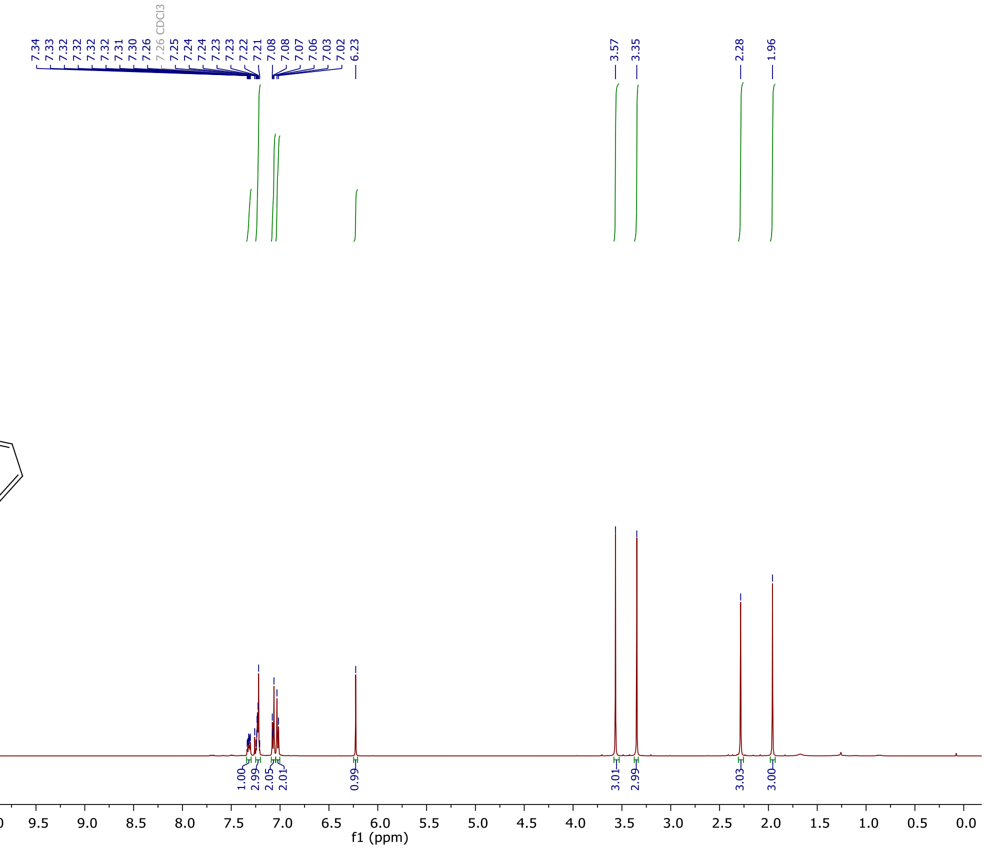

	Parameter	Value
1	Solvent	CDCl3
2	Temperature	298.1
3	Pulse Sequence	zgpg30
4	Experiment	1D
5	Number of Scans	1024
6	Receiver Gain	101.0
7	Relaxation Delay	2.0000
8	Pulse Width	10.0000
9	Spectrometer Frequency	125.77
10	Spectral Width	30120.5
11	Lowest Frequency	-2473.4
12	Nucleus	13C
13	Acquired Size	32768
14	Spectral Size	65536

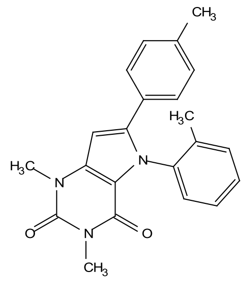

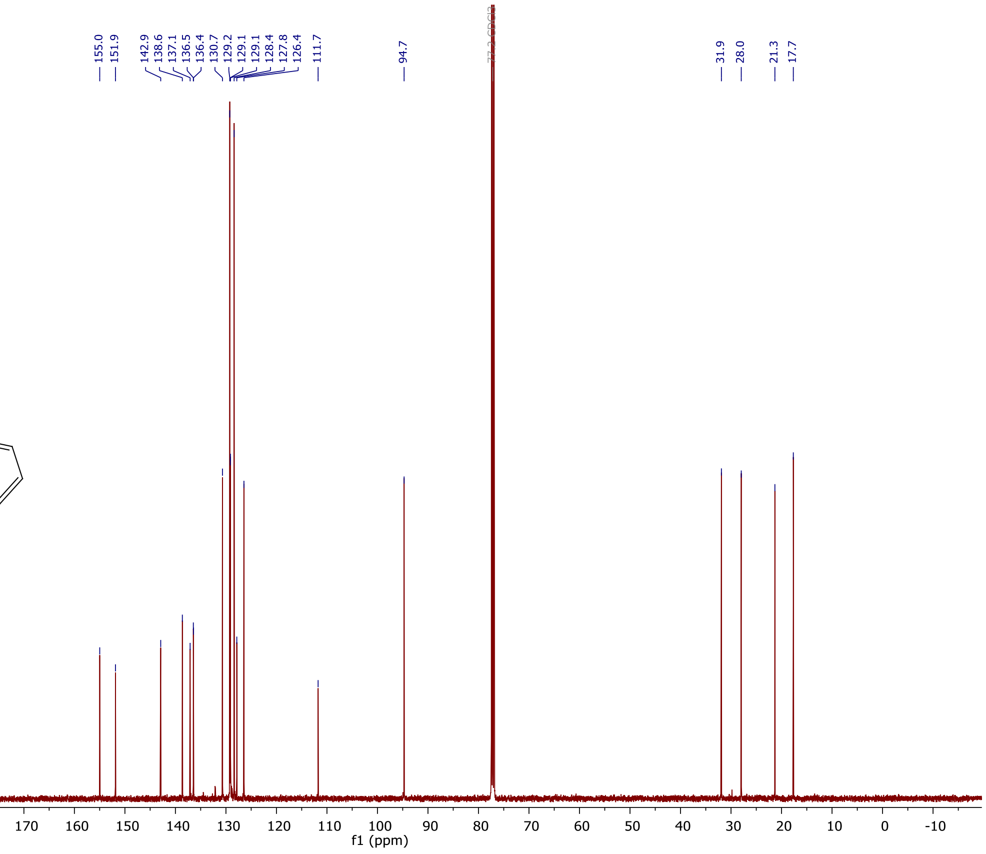

5-(2-Bromophenyl)-1,3-dimethyl-6-(*p*-tolyl)-1,5-dihydro-2*H*-pyrrolo[3,2-*d*]pyrimidine-2,4(3*H*)-dione (4f)

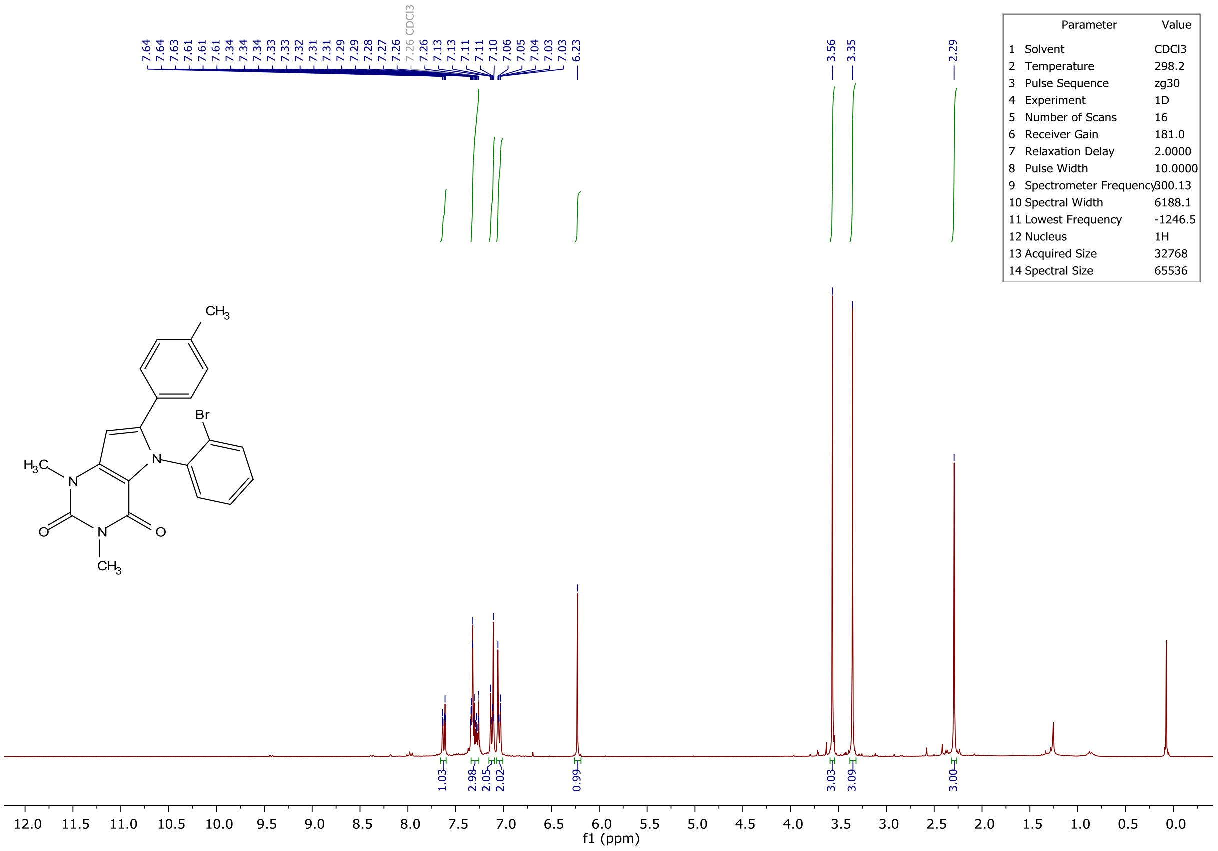

Parameter	Value
1 Solvent	CDCl3
2 Temperature	298.2
3 Pulse Sequence	zg30
4 Experiment	1D
5 Number of Scans	16
6 Receiver Gain	181.0
7 Relaxation Delay	2.0000
8 Pulse Width	10.0000
9 Spectrometer Frequency	300.13
10 Spectral Width	6188.1
11 Lowest Frequency	-1246.5
12 Nucleus	1H
13 Acquired Size	32768
14 Spectral Size	65536

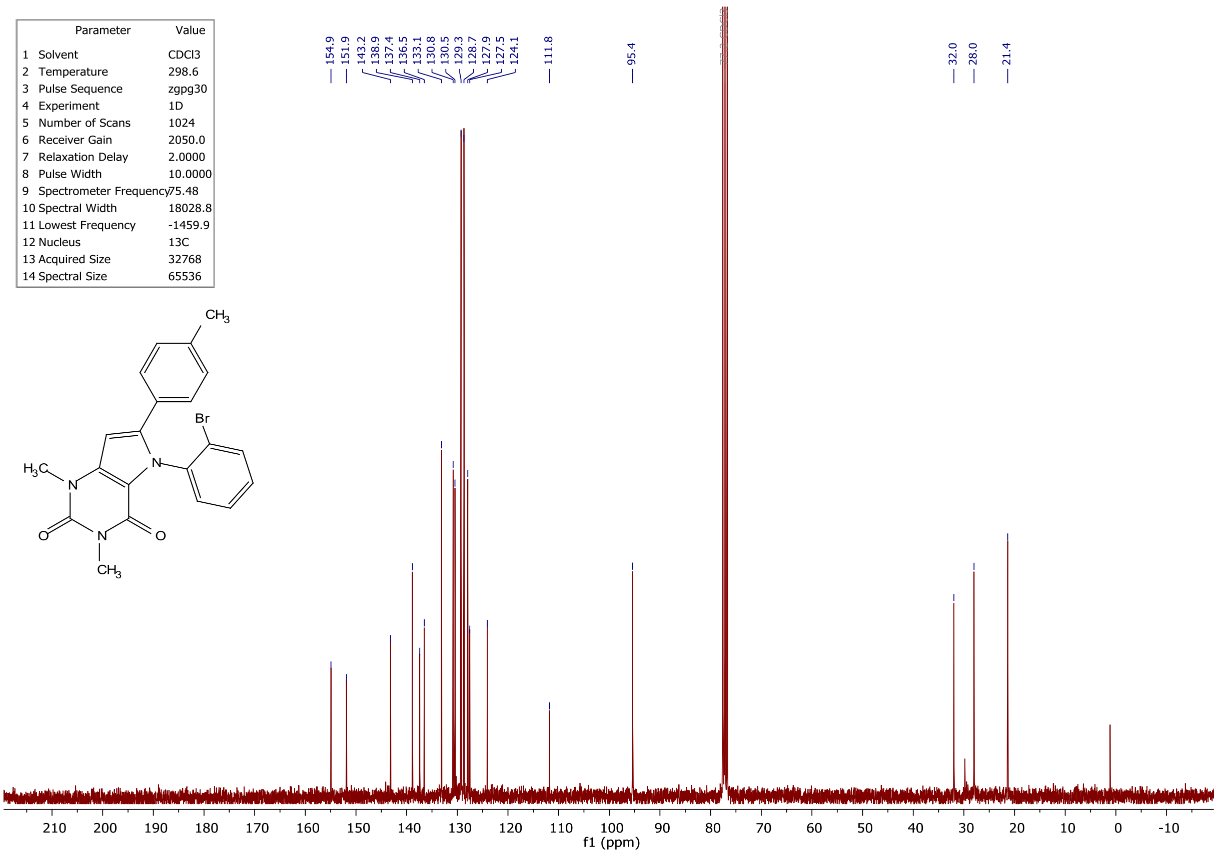

Parameter	Value
1 Solvent	CDCl3
2 Temperature	298.6
3 Pulse Sequence	zgpg30
4 Experiment	1D
5 Number of Scans	1024
6 Receiver Gain	2050.0
7 Relaxation Delay	2.0000
8 Pulse Width	10.0000
9 Spectrometer Frequency	75.48
10 Spectral Width	18028.8
11 Lowest Frequency	-1459.9
12 Nucleus	13C
13 Acquired Size	32768
14 Spectral Size	65536

1,3-Dimethyl-6-(*o*-tolyl)-5-(*p*-tolyl)-1,5-dihydro-2*H*-pyrrolo[3,2-*d*]pyrimidine-2,4(3*H*)-dione (4g)

	Parameter	Value
1	Solvent	CDCl3
2	Temperature	298.6
3	Pulse Sequence	zg30
4	Experiment	1D
5	Number of Scans	8
6	Receiver Gain	161.0
7	Relaxation Delay	2.0000
8	Pulse Width	10.0000
9	Spectrometer Frequency	300.13
10	Spectral Width	6188.1
11	Lowest Frequency	-1246.4
12	Nucleus	1H
13	Acquired Size	32768
14	Spectral Size	65536

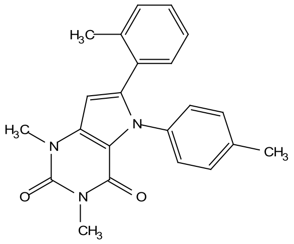

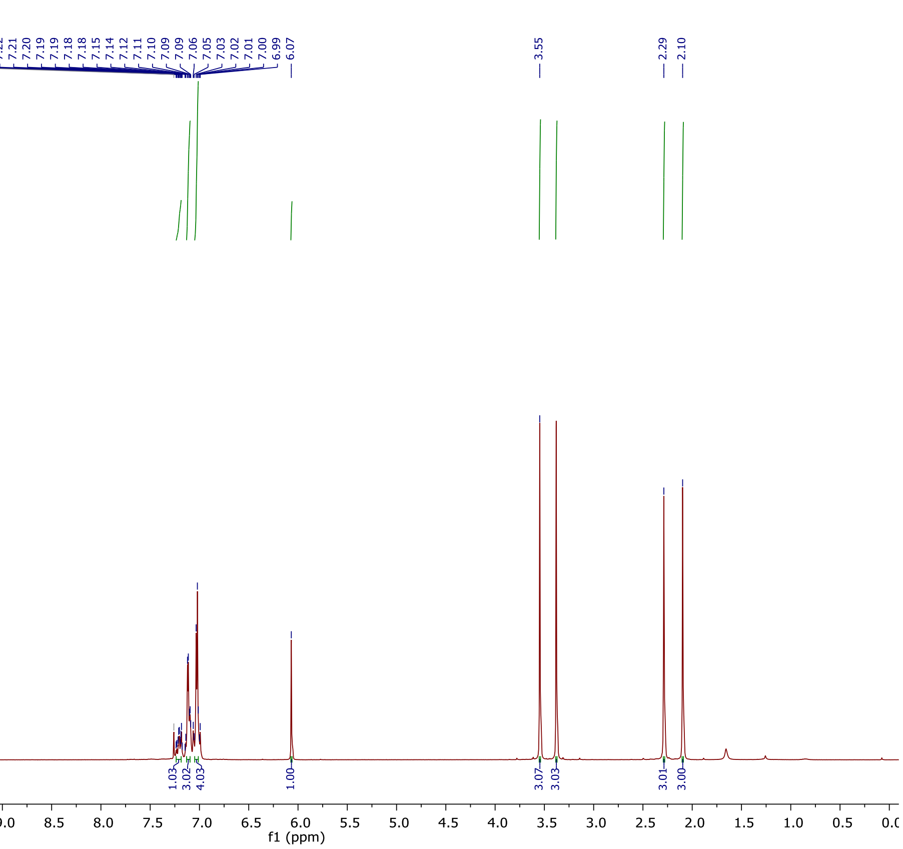

	Parameter	Value
1	Solvent	CDCl3
2	Temperature	299.3
3	Pulse Sequence	zgpg30
4	Experiment	1D
5	Number of Scans	1024
6	Receiver Gain	2050.0
7	Relaxation Delay	2.0000
8	Pulse Width	10.0000
9	Spectrometer Frequency	75.48
10	Spectral Width	18028.8
11	Lowest Frequency	-1459.7
12	Nucleus	13C
13	Acquired Size	32768
14	Spectral Size	65536

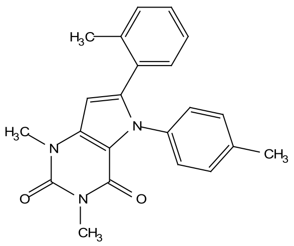

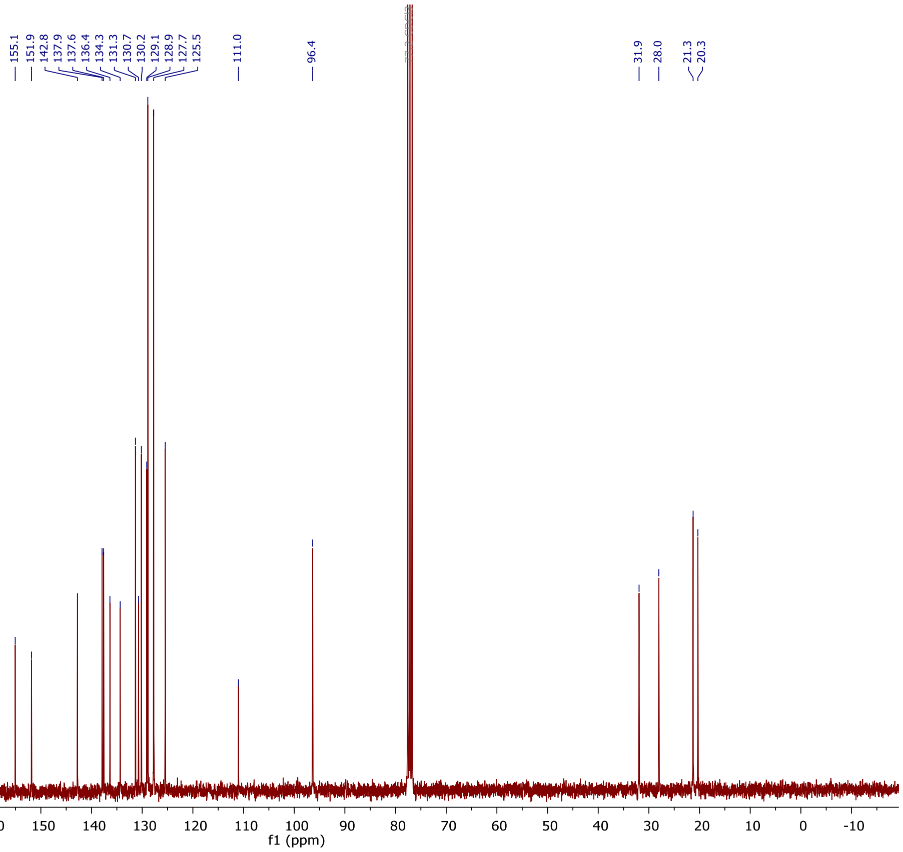

1,3-Dimethyl-6-(*m*-tolyl)-5-(*p*-tolyl)-1,5-dihydro-2*H*-pyrrolo[3,2-*d*]pyrimidine-2,4(3*H*)-dione (4h)

	Parameter	Value
1	Solvent	CDCl3
2	Temperature	298.2
3	Pulse Sequence	zg30
4	Experiment	1D
5	Number of Scans	16
6	Receiver Gain	144.0
7	Relaxation Delay	2.0000
8	Pulse Width	10.0000
9	Spectrometer Frequency	300.13
10	Spectral Width	6188.1
11	Lowest Frequency	-1246.3
12	Nucleus	1H
13	Acquired Size	32768
14	Spectral Size	65536

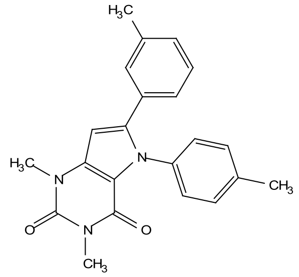

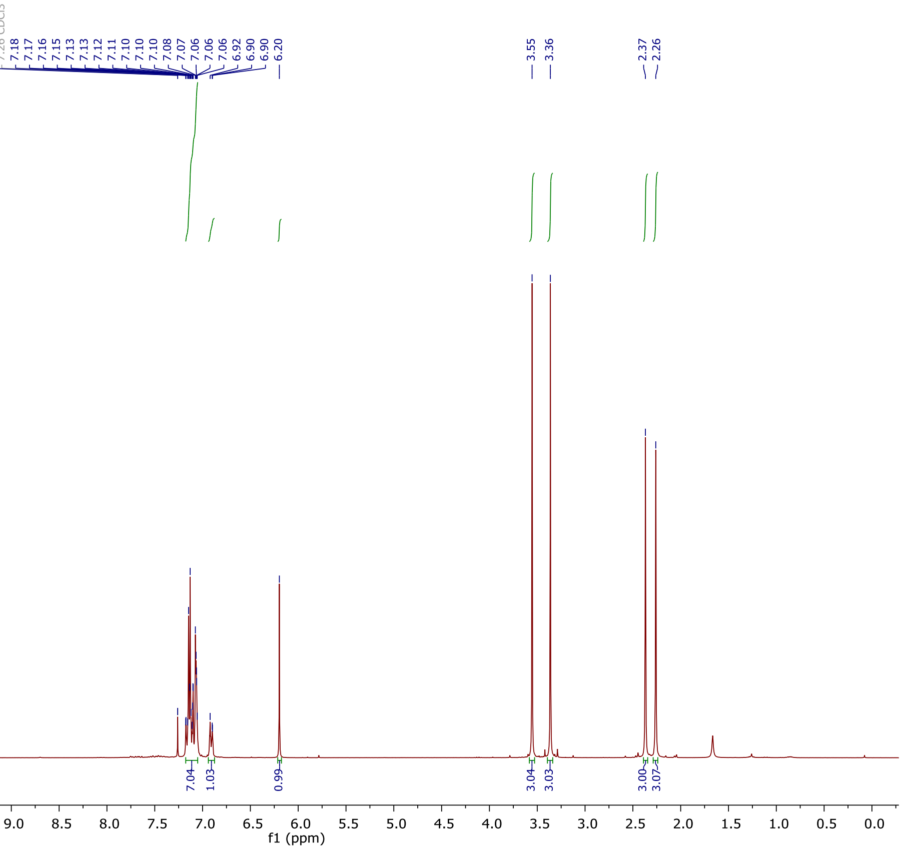

	Parameter	Value
1	Solvent	CDCl3
2	Temperature	299.2
3	Pulse Sequence	zgpg30
4	Experiment	1D
5	Number of Scans	1024
6	Receiver Gain	2050.0
7	Relaxation Delay	2.0000
8	Pulse Width	10.0000
9	Spectrometer Frequency	75.48
10	Spectral Width	18028.8
11	Lowest Frequency	-1459.8
12	Nucleus	13C
13	Acquired Size	32768
14	Spectral Size	65536

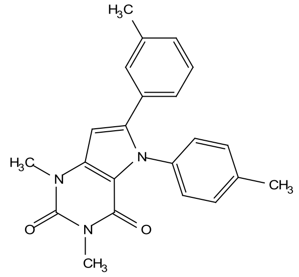

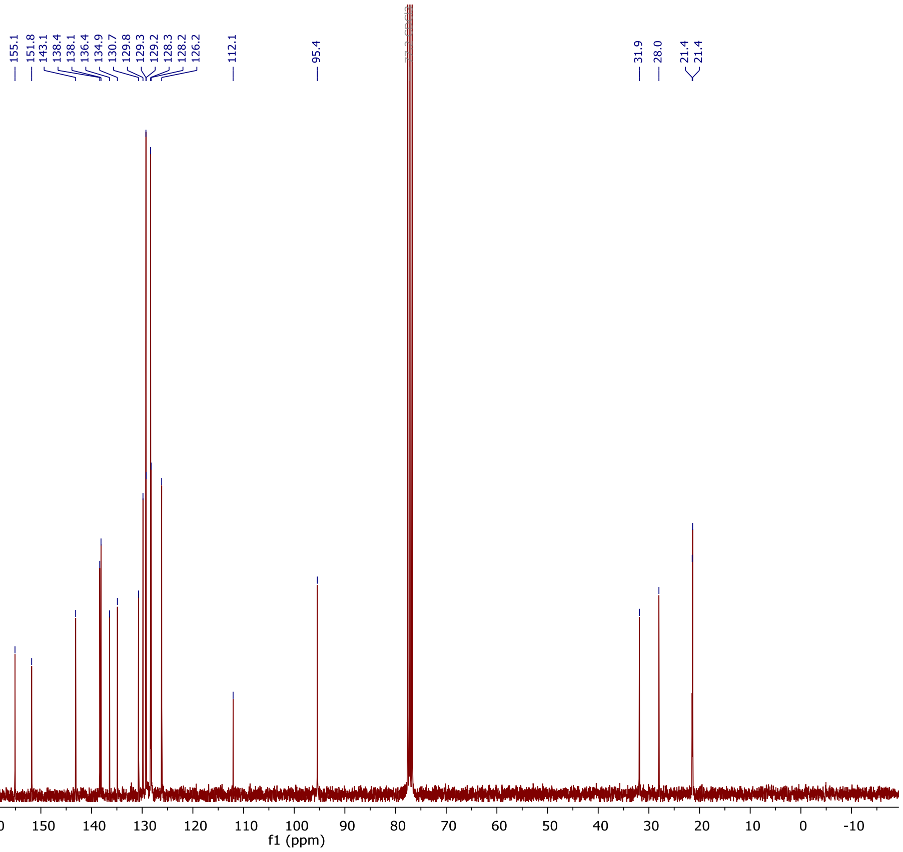

5-(4-Fluorophenyl)-1,3-dimethyl-6-(*p*-tolyl)-1,5-dihydro-2*H*-pyrrolo[3,2-*d*]pyrimidine-2,4(3*H*)-dione (4i)

	Parameter	Value
1	Solvent	CDCl3
2	Temperature	298.2
3	Pulse Sequence	zg30
4	Experiment	1D
5	Number of Scans	16
6	Receiver Gain	114.0
7	Relaxation Delay	2.0000
8	Pulse Width	10.0000
9	Spectrometer Frequency	300.13
10	Spectral Width	6188.1
11	Lowest Frequency	-1269.3
12	Nucleus	1H
13	Acquired Size	32768
14	Spectral Size	65536

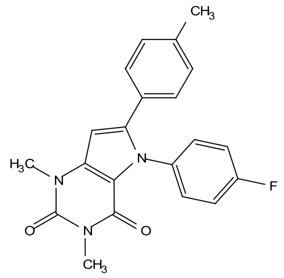

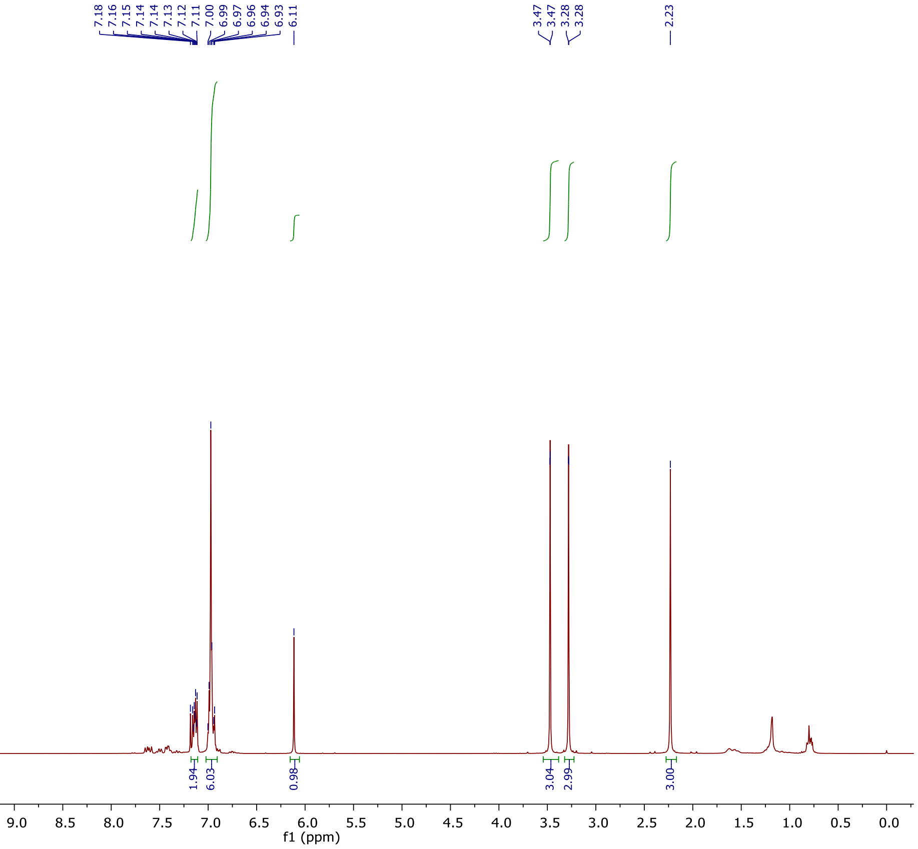

	Parameter	Value
1	Solvent	CDCl3
2	Temperature	298.2
3	Pulse Sequence	zgfhigqn
4	Experiment	1D
5	Number of Scans	64
6	Receiver Gain	2050.0
7	Relaxation Delay	1.0000
8	Pulse Width	10.0000
9	Spectrometer Frequency	282.38
10	Spectral Width	66964.3
11	Lowest Frequency	-61722.4
12	Nucleus	19F
13	Acquired Size	65536
14	Spectral Size	131072

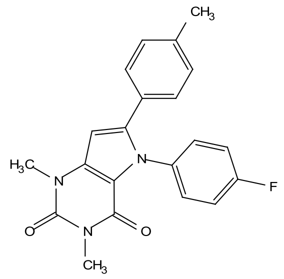

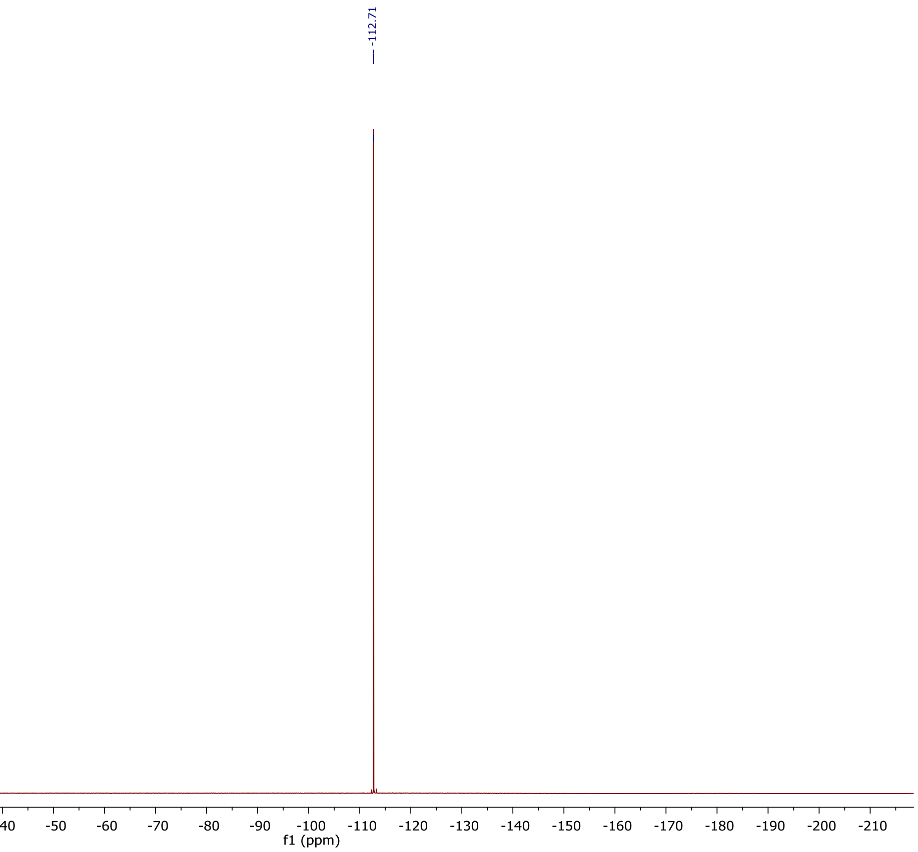

	Parameter	Value
1	Solvent	CDCl3
2	Temperature	299.6
3	Pulse Sequence	zgpg30
4	Experiment	1D
5	Number of Scans	1024
6	Receiver Gain	2050.0
7	Relaxation Delay	2.0000
8	Pulse Width	10.0000
9	Spectrometer Frequency	75.48
10	Spectral Width	18028.8
11	Lowest Frequency	-1460.7
12	Nucleus	13C
13	Acquired Size	32768
14	Spectral Size	65536

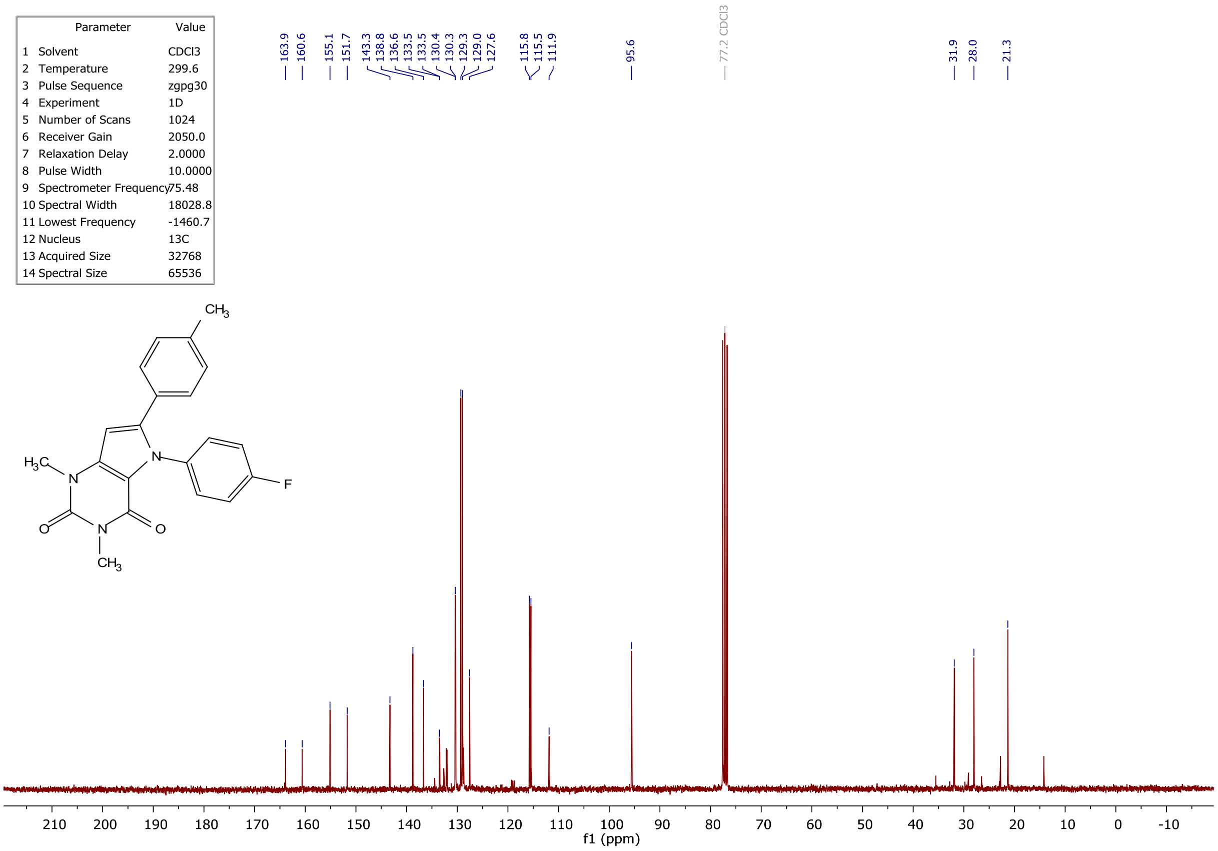

1,3-Dimethyl-6-(*p*-tolyl)-5-(4-(trifluoromethyl)phenyl)-1,5-dihydro-2*H*-pyrrolo[3,2-*d*]-pyrimidine-2,4(3*H*)-dione (4j)

	Parameter	Value
1	Solvent	CDCl3
2	Temperature	298.2
3	Pulse Sequence	zg30
4	Experiment	1D
5	Number of Scans	16
6	Receiver Gain	144.0
7	Relaxation Delay	2.0000
8	Pulse Width	10.0000
9	Spectrometer Frequency	300.13
10	Spectral Width	6188.1
11	Lowest Frequency	-1246.3
12	Nucleus	1H
13	Acquired Size	32768
14	Spectral Size	65536

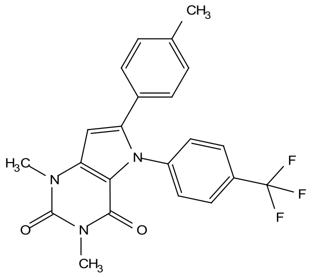

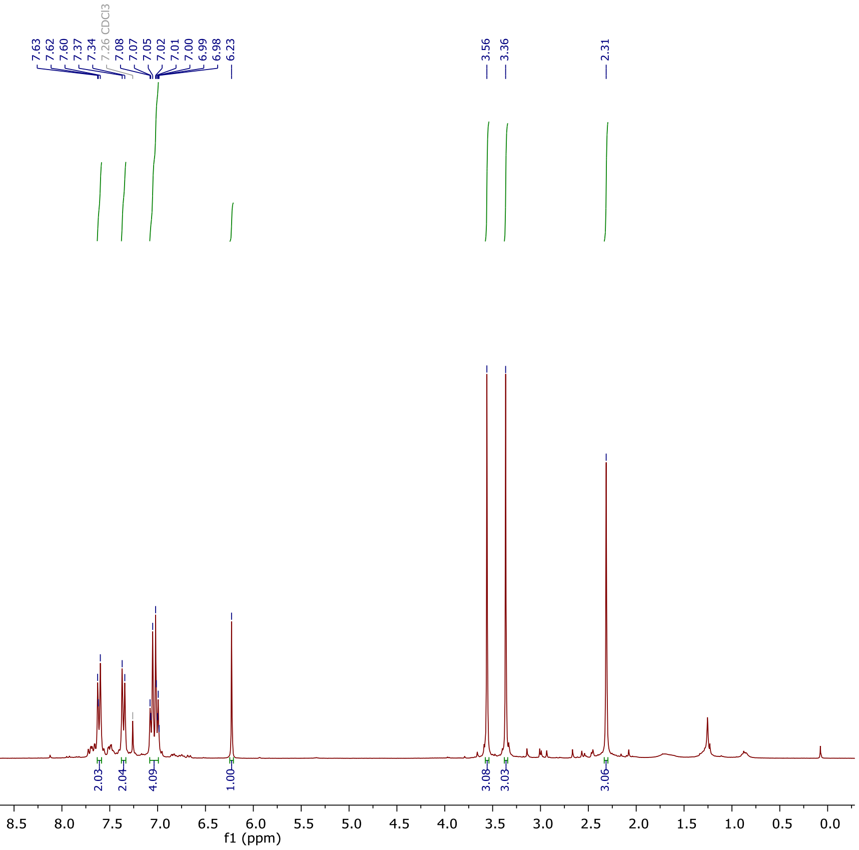

	Parameter	Value
1	Solvent	CDCl3
2	Temperature	298.3
3	Pulse Sequence	zgfhigqn
4	Experiment	1D
5	Number of Scans	64
6	Receiver Gain	2050.0
7	Relaxation Delay	1.0000
8	Pulse Width	10.0000
9	Spectrometer Frequency	282.38
10	Spectral Width	66964.3
11	Lowest Frequency	-61722.4
12	Nucleus	19F
13	Acquired Size	65536
14	Spectral Size	131072

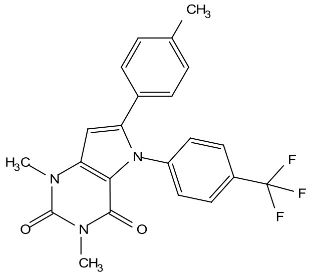

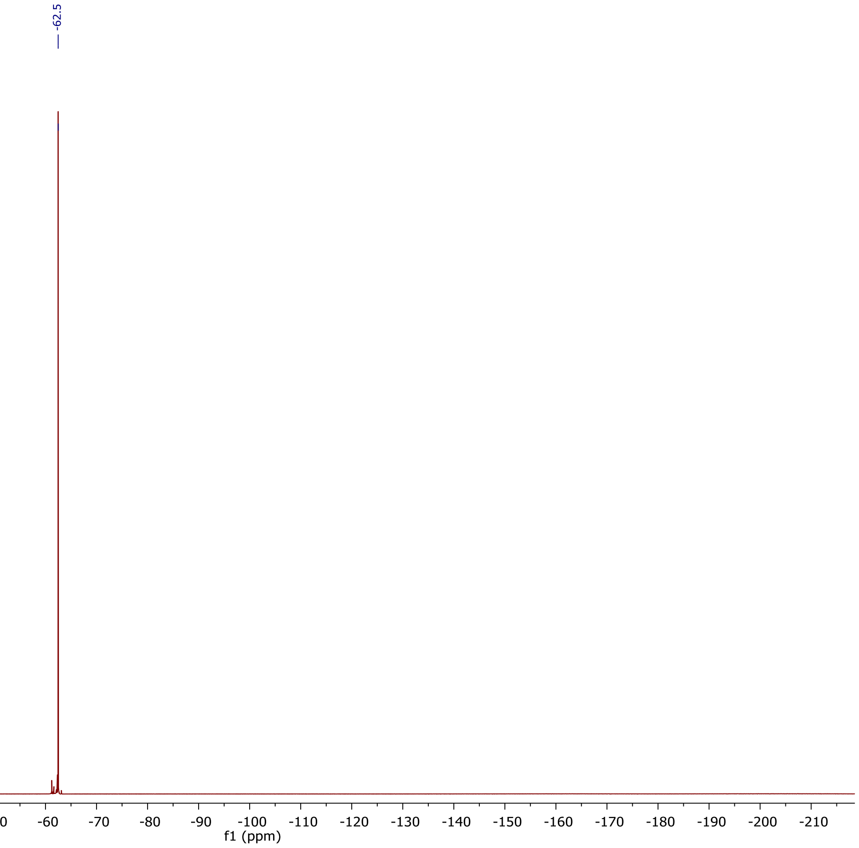

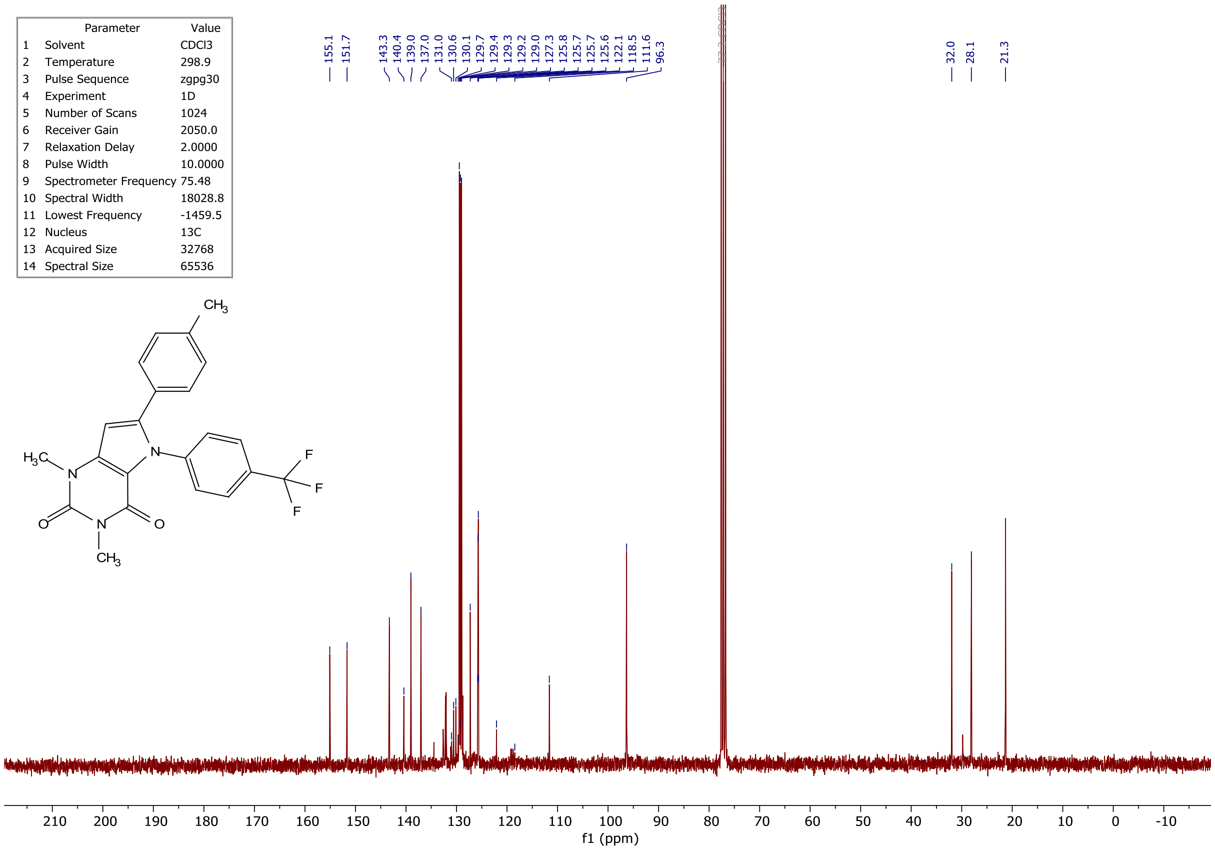
Parameter Value
1 Solvent CDCl3
2 Temperature 298.9
3 Pulse Sequence zgpg30
4 Experiment 1D
5 Number of Scans 1024
6 Receiver Gain 2050.0
7 Relaxation Delay 2.0000
8 Pulse Width 10.0000
9 Spectrometer Frequency 75.48
10 Spectral Width 18028.8
11 Lowest Frequency -1459.5
12 Nucleus 13C
13 Acquired Size 32768
14 Spectral Size 65536
155.1
151.7
143.3
140.4
139.0
137.0
131.0
130.6
130.1
129.7
129.4
129.3
129.2
129.0
127.3
125.8
125.7
125.6
122.1
118.5
111.6
96.3
32.0
28.1
21.3
CH3
H3C
N
N
N
O
O
CH3
F
F
F
210 200 190 180 170 160 150 140 130 120 110 100 90 80 70 60 50 40 30 20 10 0 -10
f1 (ppm)

6-(4-(Dimethylamino)phenyl)-1,3-dimethyl-5-(*p*-tolyl)-1,5-dihydro-2*H*-pyrrolo[3,2-*d*]pyrimidine-2,4(3*H*)-dione (4k)

	Parameter	Value
1	Solvent	CDCl3
2	Temperature	298.6
3	Pulse Sequence	zg30
4	Experiment	1D
5	Number of Scans	8
6	Receiver Gain	228.0
7	Relaxation Delay	2.0000
8	Pulse Width	10.0000
9	Spectrometer Frequency	300.13
10	Spectral Width	6188.1
11	Lowest Frequency	-1246.6
12	Nucleus	1H
13	Acquired Size	32768
14	Spectral Size	65536

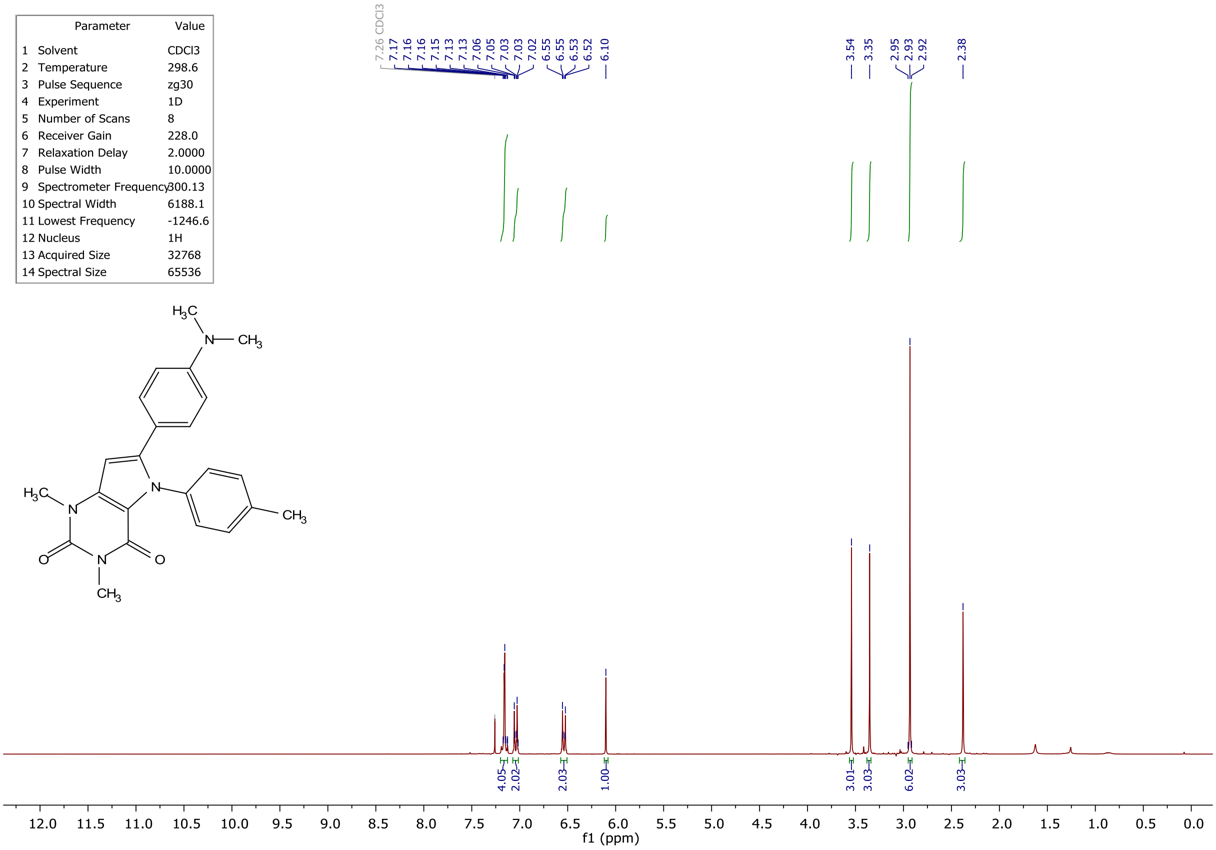

	Parameter	Value
1	Solvent	CDCl3
2	Temperature	298.7
3	Pulse Sequence	zgpg30
4	Experiment	1D
5	Number of Scans	1024
6	Receiver Gain	2050.0
7	Relaxation Delay	2.0000
8	Pulse Width	10.0000
9	Spectrometer Frequency	75.48
10	Spectral Width	18028.8
11	Lowest Frequency	-1468.1
12	Nucleus	13C
13	Acquired Size	32768
14	Spectral Size	65536

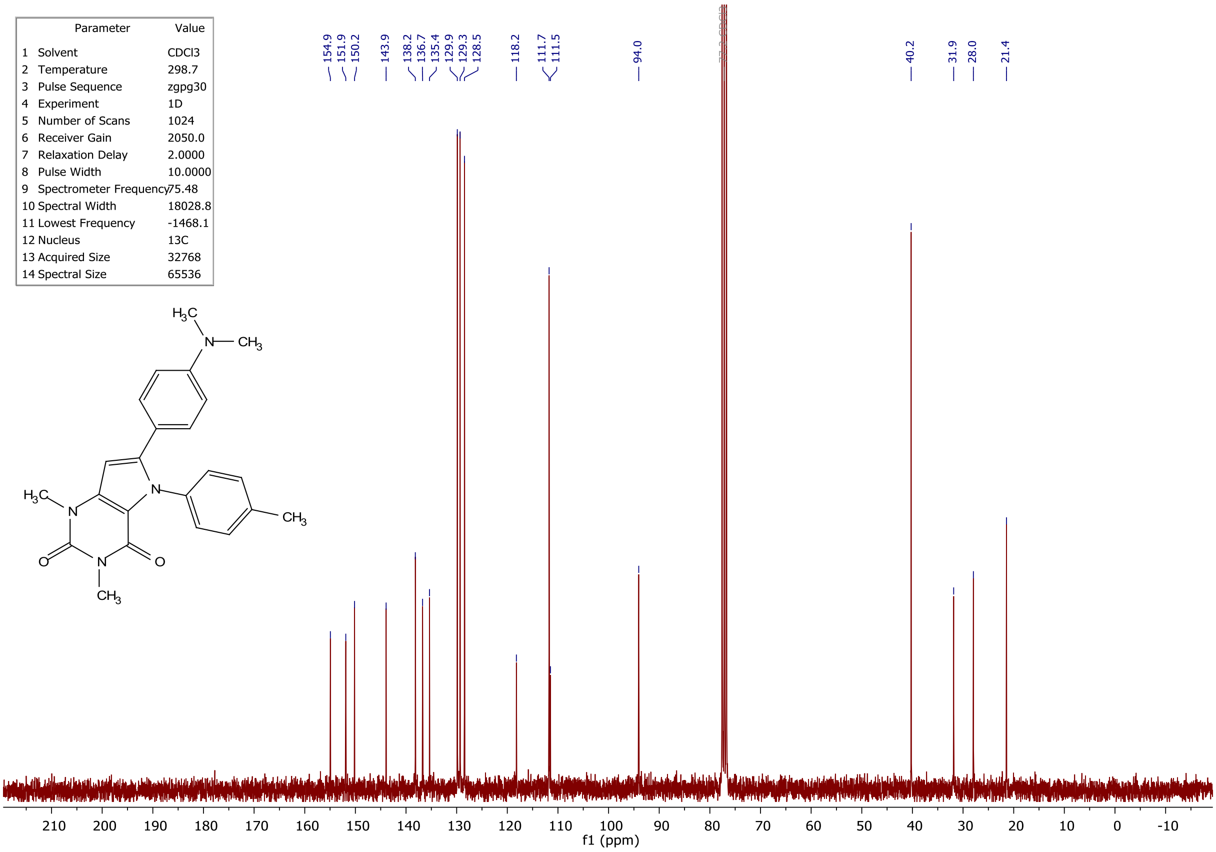

6-(4-(Dimethylamino)phenyl)-1,3-dimethyl-5-(4-(trifluoromethyl)phenyl)-1,5-dihydro-2*H*-pyrrolo[3,2-*d*]pyrimidine-2,4(3*H*)-dione (4l)

	Parameter	Value
1	Solvent	CDCl3
2	Temperature	298.2
3	Pulse Sequence	zg30
4	Experiment	1D
5	Number of Scans	16
6	Receiver Gain	161.0
7	Relaxation Delay	2.0000
8	Pulse Width	10.0000
9	Spectrometer Frequency	300.13
10	Spectral Width	6188.1
11	Lowest Frequency	-1246.5
12	Nucleus	1H
13	Acquired Size	32768
14	Spectral Size	65536

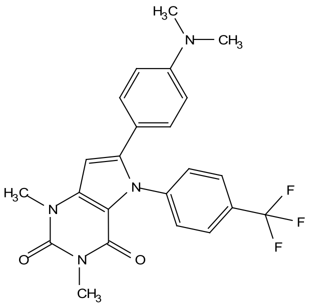

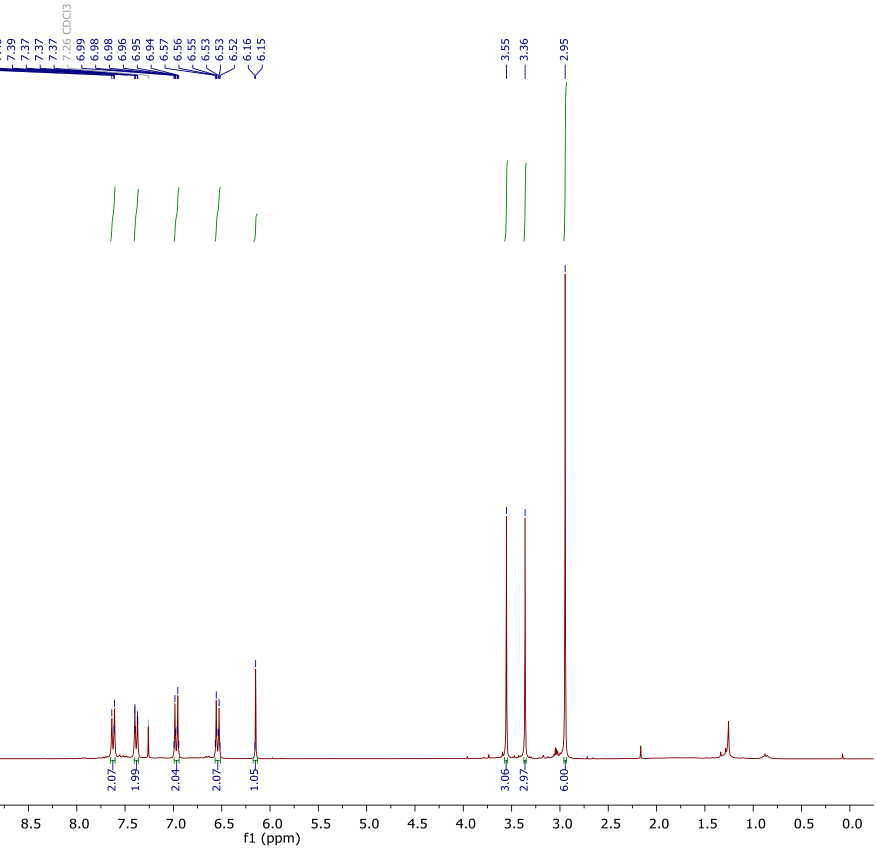

	Parameter	Value
1	Solvent	CDCl3
2	Temperature	298.2
3	Pulse Sequence	zgfhigqn
4	Experiment	1D
5	Number of Scans	64
6	Receiver Gain	2050.0
7	Relaxation Delay	1.0000
8	Pulse Width	10.0000
9	Spectrometer Frequency	282.38
10	Spectral Width	66964.3
11	Lowest Frequency	-61722.4
12	Nucleus	19F
13	Acquired Size	65536
14	Spectral Size	131072

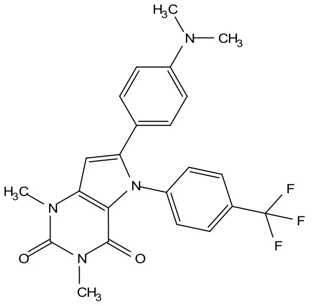

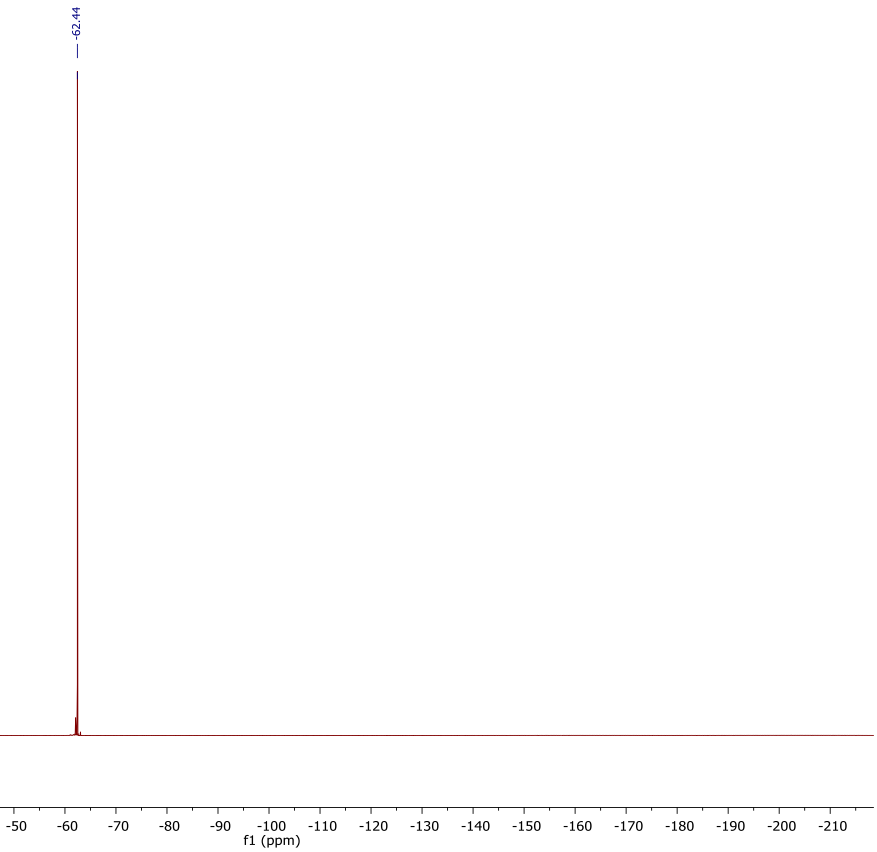

	Parameter	Value
1	Solvent	CDCl3
2	Temperature	298.7
3	Pulse Sequence	zgpg30
4	Experiment	1D
5	Number of Scans	1024
6	Receiver Gain	2050.0
7	Relaxation Delay	2.0000
8	Pulse Width	10.0000
9	Spectrometer Frequency	75.48
10	Spectral Width	18028.8
11	Lowest Frequency	-1459.3
12	Nucleus	13C
13	Acquired Size	32768
14	Spectral Size	65536

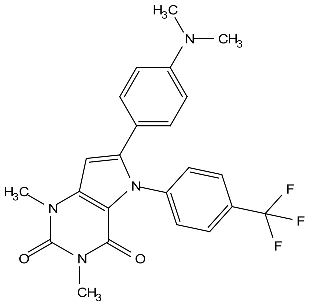

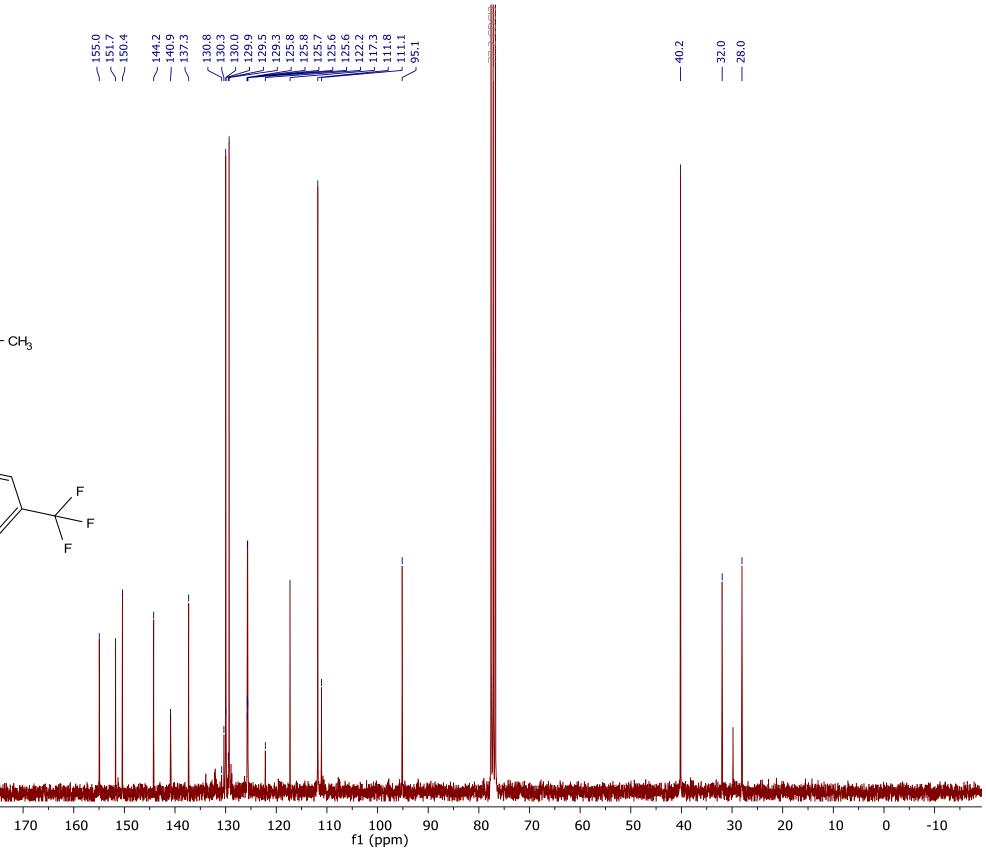

1,3-Dimethyl-6-(thiophen-3-yl)-5-(*p*-tolyl)-1,5-dihydro-2*H*-pyrrolo[3,2-*d*]pyrimidine-2,4(3*H*)-dione (4m)

	Parameter	Value
1	Solvent	CDCl3
2	Temperature	298.6
3	Pulse Sequence	zg30
4	Experiment	1D
5	Number of Scans	8
6	Receiver Gain	256.0
7	Relaxation Delay	2.0000
8	Pulse Width	10.0000
9	Spectrometer Frequency	300.13
10	Spectral Width	6188.1
11	Lowest Frequency	-1268.1
12	Nucleus	1H
13	Acquired Size	32768
14	Spectral Size	65536

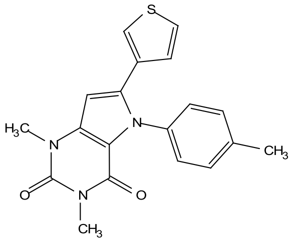

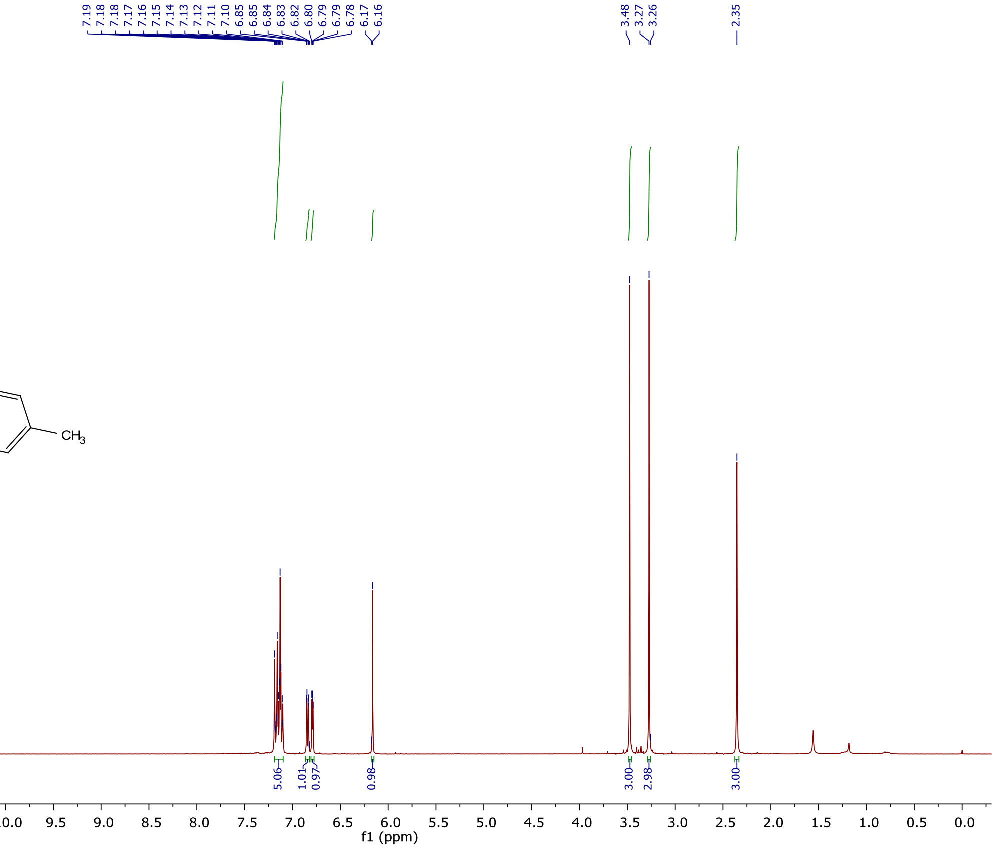

	Parameter	Value
1	Solvent	CDCl3
2	Temperature	299.6
3	Pulse Sequence	zgpg30
4	Experiment	1D
5	Number of Scans	1024
6	Receiver Gain	2050.0
7	Relaxation Delay	2.0000
8	Pulse Width	10.0000
9	Spectrometer Frequency	75.48
10	Spectral Width	18028.8
11	Lowest Frequency	-1458.8
12	Nucleus	13C
13	Acquired Size	32768
14	Spectral Size	65536

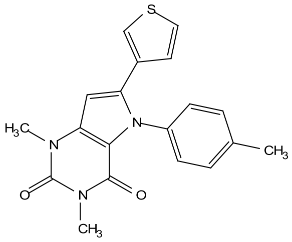

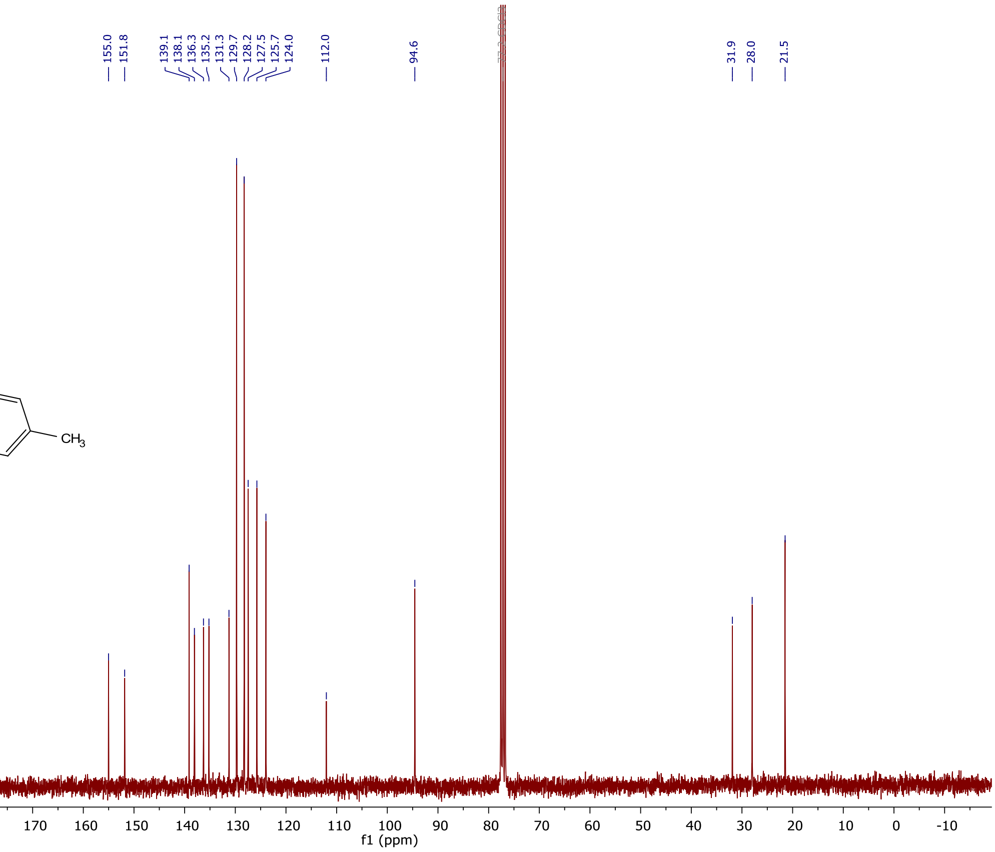

References

[1] Abreu, R. M. F. de; Brockmann, T.; Villinger, A.; Ehlers, P.; Langer, P. *Beilstein J. Org. Chem.* **2024,** *20*, 898–911. doi:10.3762/bjoc.20.80
